# A novel photosynthetic biologic topical gel for enhanced localized hyperoxygenation augments wound healing in peripheral artery disease

**DOI:** 10.1038/s41598-022-14085-1

**Published:** 2022-06-15

**Authors:** Yuanjia Zhu, Jinsuh Jung, Shreya Anilkumar, Sidarth Ethiraj, Sarah Madira, Nicholas A. Tran, Danielle M. Mullis, Kerriann M. Casey, Sabrina K. Walsh, Charles J. Stark, Akshay Venkatesh, Alexander Boakye, Hanjay Wang, Y. Joseph Woo

**Affiliations:** 1 Department of Cardiothoracic Surgery, Stanford University, Stanford, CA USA; 2 Department of Bioengineering, Stanford University, Stanford, CA USA; 3 Department of Comparative Medicine, Stanford University, Stanford, CA USA; 4 Department of Cardiothoracic Surgery, Falk Cardiovascular Research Center, Stanford University School of Medicine, 300 Pasteur Drive, Stanford, CA 94305 USA

**Keywords:** Cardiovascular diseases, Vascular diseases

## Abstract

Peripheral artery disease and the associated ischemic wounds are substantial causes of global morbidity and mortality, affecting over 200 million people worldwide. Although advancements have been made in preventive, pharmacologic, and surgical strategies to treat this disease, ischemic wounds, a consequence of end-stage peripheral artery disease, remain a significant clinical and economic challenge. *Synechococcus elongatus* is a cyanobacterium that grows photoautotrophically and converts carbon dioxide and water into oxygen. We present a novel topical biologic gel containing *S. elongatus* that provides oxygen via photosynthesis to augment wound healing by rescuing ischemic tissues caused by peripheral artery disease. By using light rather than blood as a source of energy, our novel topical therapy significantly accelerated wound healing in two rodent ischemic wound models. This novel topical gel can be directly translated to clinical practice by using a localized, portable light source without interfering with patients’ daily activities, demonstrating potential to generate a paradigm shift in treating ischemic wounds from peripheral artery disease. Its novelty, low production cost, and ease of clinical translatability can potentially impact the clinical care for millions of patients suffering from peripheral arterial disease.

## Introduction

Peripheral artery disease (PAD) is a substantial cause of global morbidity and mortality, affecting over 200 million people worldwide^1^. This number is anticipated to rise due to increasing life expectancy in developed countries^2,3^. Although advances have been made over the past several decades in preventive, pharmacologic, and surgical strategies to treat PAD, it still remains underdiagnosed and undertreated^4,5^. In end-stage PAD, ischemic wounds are common, resulting in significant tissue loss^6,7^. Every year, over 50 billion U.S. dollars were spent on wound care in the U.S., representing a substantial portion of the annual healthcare expenditure^8–10^. Additionally, these ischemic wounds are the main cause of amputations with poor patient survival if revascularization is not performed^11^.

*Synechococcus elongatus* is a naturally occurring unicellular cyanobacterium that grows photoautotrophically^12^. Cyanobacteria are among the primary producers of environmental oxygen by harnessing the power from a broad wavelength spectrum and converting carbon dioxide and water into oxygen^13^. A broad range of applications exist for cyanobacteria including the traditional use as a model for circadian rhythms and biofuel production^14–16^, to more recent advancements such as using its photosynthetic nature to increase tissue oxygenation and rescue myocardium from acute ischemia^12^. We hypothesized that a novel biologic gel containing *S. elongatus* could provide oxygen via photosynthesis to ischemic tissue caused by PAD, therefore maintaining tissue metabolism and accelerating wound healing. This innovative gel shows great potential as a highly effective, affordable topical treatment for ischemic wounds from PAD and could potentially greatly impact the clinical care for this challenging population.

## Results

### Hyaluronic acid gel using BG11 medium supports high *S. elongatus* viability and oxygen production

**Figure 1:**
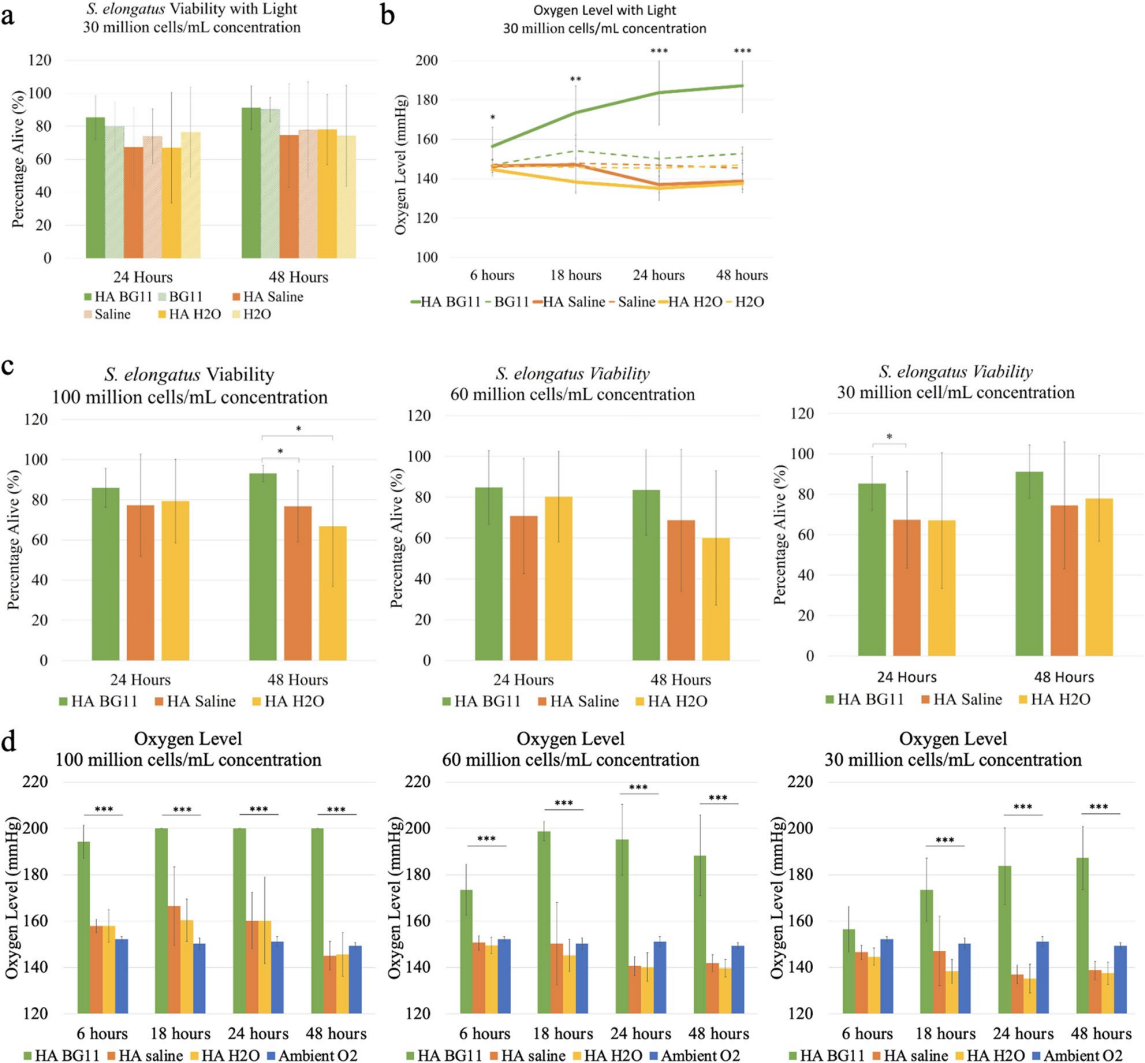
Effect of hyaluronic acid on S. elongatus viability and oxygen production with light exposure. (**a**) S. elongatus viability at 30 million cells/mL in BG11 medium, normal saline, and H_2_O with and without hyaluronic acid were similar between the groups and after 24 compared to 48 h of incubation. Statistical analyses were performed using the student t test. (**b**) At 30 million cells/mL, hyaluronic acid gel in BG11 with S. elongatus under light incubation was associated with the highest oxygen level at 6, 18, 24, and 48 h compared to the other groups. Hyaluronic acid gel in BG11 compared to BG11 only with S. elongatus, was associated with significantly higher oxygen level at all time points based on post-hoc correction (p < 0.009). The addition of hyaluronic acid to normal saline or water was associated with lower oxygen level at 18, 24, and 48 h (p < 0.004 and p < 0.002, respectively) based on post-hoc correction. Statistical analyses shown in the figure were performed using the analysis of variance at each time point. (**c**) S. elongatus viability at 30, 60, or 100 million cells/mL concentration remained similar within the same gel composition at 24 and 48 h. S. elongatus viability was generally higher when incubated in the hyaluronic acid in BG11 compared to other gel compositions. Statistical analyses were performed using the student t test. (**d**) Oxygen production was consistently higher in the hyaluronic acid gel in BG11 at 30, 60, and 100 million cells/mL concentration compared to hyaluronic acid in normal saline (p < 0.007, p < 0.0001, and p < 0.0001, respectively) and hyaluronic acid in water (p < 0.002, p < 0.0001, and p < 0.0001, respectively) based on post-hoc correction. Data presented as mean ± standard deviation. A total of 10 samples were tested for each gel composition for each treatment at each time point. Statistics shown in (**d**) were resulted from the analysis of variance. * Indicates p < 0.05, ** indicates p < 0.01, and *** indicates p < 0.001.

To evaluate the effect of hyaluronic acid (HA) on *S. elongatus* viability and oxygen production, *S. elongatus* was inoculated at a concentration of 30 million cells/mL in BG11 medium, sterile normal saline, and sterile H_2_O, each medium having conditions with and without incorporating HA. There was no statistical difference in *S. elongatus* viability in conditions with versus without HA at 24 versus 48 h (Figs. 1a, S1a, Table S1). HA gel in BG11 with *S. elongatus* in light was associated with the highest oxygen level at 6, 18, 24, and 48 h after inoculation compared to other compositions (Fig. 1b, Table S2). Without light, the addition of HA was associated with lower levels of oxygen at 24 and 48 h (Fig. S1b, Table S2).

### High concentration of *S. elongatus* provides sustained maximal oxygen production

To investigate the effect of *S. elongatus* inoculation concentrations, *S. elongatus* viability was assessed when inoculated in HA gel in BG11, saline, and H_2_O at 30, 60, and 100 million cells/mL (Table S3). No statistical difference in *S. elongatus* viability was observed among different concentrations within the same gel composition and at 24 versus 48 h in light, except for HA gel in H_2_O that was associated with decreased *S. elongatus* viability at 60 million cells/mL from 24 to 48 h. At 100 million cells/mL, HA gel in BG11 was associated with the highest *S. elongatus* viability at 48 h compared to that of HA gel in saline and H_2_O (Fig. 1c). Additionally, HA gel in BG11 consistently outperformed HA gel in saline and H_2_O in oxygen level (Fig. 1d, Table S4). HA gel in BG11 with 100 million *S. elongatus*/mL inoculated reached the highest measurable oxygen level within 18 h and sustained for 48 h (Fig. 1d). *S. elongatus* viability and oxygen level without light are shown in Fig. S1c,d. Given the high *S. elongatus* viability and oxygen production level, HA gel in BG11 with 100 million *S. elongatus*/mL was chosen as the final composition for the novel biologic gel for the remainder of the studies.

### The novel biologic gel has prolonged shelf life

To evaluate the shelf life of our novel biologic gel, we performed *S. elongatus* viability testing while incubating the biologic gel in light for 5 weeks (Table S5). As shown in Fig. S2, a high *S. elongatus* viability of above 80% was observed for 2 weeks in the novel biologic gel without requiring medium addition.

### The novel biologic gel improved fibroblast survival and migration during hypoxia

**Figure 2:**
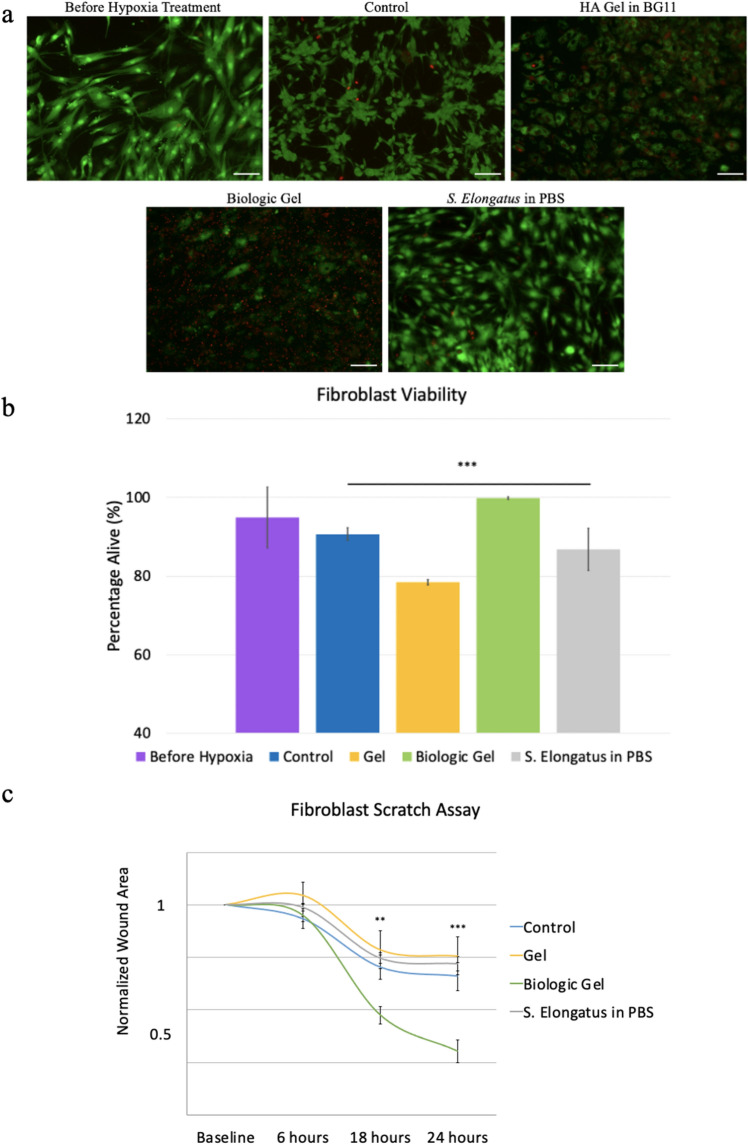
Human dermal fibroblast viability and migration under hypoxia after biologic gel treatment. (**a**,**b**) Before hypoxia treatment under the normoxic condition, high fibroblast viability with normal morphology was observed. After 24 h in the serum-starved, 2% O_2_ hypoxia condition, a decrease in fibroblast viability was noted with altered cellular morphology. Treatment with hyaluronic acid in BG11 further decreased fibroblast viability compared to control (p = 0.04), whereas treatment with the novel biologic gel and S. elongatus in PBS in light greatly restored cellular morphology compared to baseline in the normoxic condition. Treatment with the novel biologic gel significantly improved fibroblast viability compared to control (p = 0.02). No difference was observed in fibroblast viability between control and treatment using S. elongatus in PBS in light (p = 0.34). A total of 10 samples were measured for each treatment group. Data presented as mean ± standard deviation. Green = alive; red = dead. S. elongatus was stained red irrespective of its viability using this mammalian live/dead assay. Scale bar = 100 µm. (**c**) Fibroblast migration demonstrated by wound healing in a scratch assay showed the smallest normalized wound area at 18 and 24 h after treatment using the novel biologic gel compared control (p = 0.009 and p = 0.002, respectively). No difference was observed in the normalized wound area at 6 h among the different treatment groups. At 18 and 24 h, treatment using hyaluronic acid in BG11 and E. elongatus in PBS did not result in any difference in normalized wound area compared to control. The control group refers to cells that received PBS treatment under the hypoxia condition. A total of 5 samples were measured for each treatment group. The error bars represent standard error. Statistical analysis was performed using the analysis of variance (ANOVA) with post-hoc correction. Statistics shown on the figure represent the results from ANOVA.* Indicates p < 0.05, ** indicates p < 0.01, and *** indicates p < 0.001.

To assess whether the novel biologic gel can enhance mammalian cell survival in vitro, we measured human dermal fibroblast (HDFa) viability in a serum-starved, hypoxic condition in light for 24 h after treatment (Fig. 2a,b, Table S6). HDFa cells that received novel biologic gel treatment demonstrated the most superior cell viability with more normal morphology compared to cells that received other treatments. There was no statistical difference in HDFa cell viability between cells that received the novel biologic gel and cells at baseline under the normoxic condition. HDFa cells that received the *S. elongatus* in PBS demonstrated restored cellular morphology but cell viability remained inferior compared to those treated with the novel biologic gel. To assess whether the novel biologic gel can enhance mammalian cell migration in vitro, HDFa migration was performed using scratch assay in a hypoxic condition in light for 24 h after treatment (Figs. 2c, S3). HDFa cells that received the novel biologic gel treatment demonstrated the smallest normalized wound area compared to the other treatment groups at 18 and 24 h after treatment.

### Treatment with the novel biologic gel immediately increased tissue oxygenation during acute ischemia in the PAD burn wound model

Next, we evaluated the effect of the novel biologic gel on tissue oxygenation in vivo by measuring oxygen levels at baseline, 10 min after ischemia, as well as 10 and 20 min after treatment (control without treatment, HA gel in BG11, and novel biologic gel in light or dark) on the tarsal wounds in the rodent PAD burn wound model (Fig. S4a). Baseline tissue oxygen levels were similar among all four groups and significantly decreased after the ligation of femoral artery. In light, the novel biologic gel treatment resulted in a sevenfold increase in tissue oxygen levels after 10 and 20 min of treatment compared to that of the ischemic condition. By comparison, wounds treated with HA gel in BG11, biologic gel in dark, and control (no treatment) showed only 1.5- and 2.6-, 1.8- and 1.8-, as well as 1.8- and 1.7-fold increase in tissue oxygen levels after 10 and 20 min of treatment, respectively.

### The novel biologic gel treatment significantly improved wound healing in the PAD burn wound model

We evaluated the effect of novel biologic gel on wound healing in the rodent PAD burn wound model. Specifically, we investigated the wound healing speed by measuring unhealed wound area and the time needed for complete wound healing. Wounds that received the novel biologic gel in light demonstrated the smallest unhealed wound area after 3, 5, and 7 days of daily treatment compared to wounds that received other treatments (Fig. S4b). Overall, wounds that received the novel biologic gel treatment in light healed the fastest with an average time to heal of 12 ± 2.7 days compared to 15.8 ± 2.6 days without treatment (*p* = 0.03), 16.3 ± 2.9 days with HA gel in BG11 treatment, and 16.8 ± 3.4 days with biologic gel treatment in dark (Fig. S4c). No hypertrophic scar was observed in any animals.

**Figure 3:**
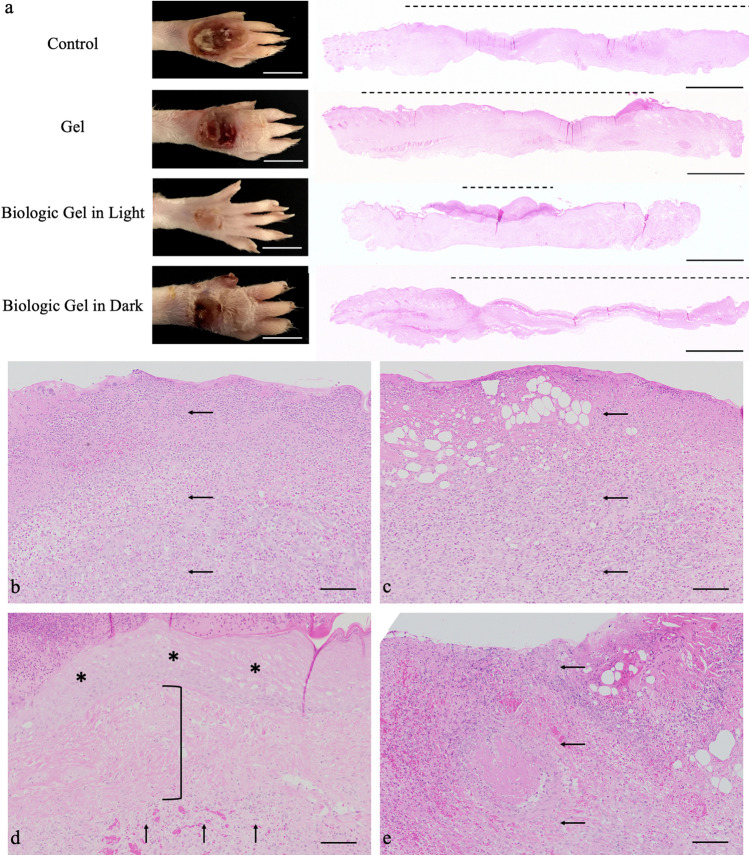
Gross and histopathological evaluation of tarsal wounds after 7 days of treatment in a rodent peripheral arterial disease burn wound model. (**a**) Gross tarsal lesions after 7 days of daily treatment showed smaller wound area and less ulceration in the wound that received the novel biologic gel treatment in light compared to the wounds that received other treatment. Scale bar = 1 cm. Hematoxylin and eosin stain of longitudinal sections of the tarsal lesions showed that wounds that were treated with biologic gel in light generally had less extensive ulceration (dashed lines) than wounds that received other treatment. Scale bar = 2 mm. (**b**–**e**) Hematoxylin and eosin stain of cross sections of the tarsal lesions under higher magnification showed that wounds that were treated with biologic gel in light (**d**) generally had milder lesions than wounds that were not treated (**b**) or treated with gel (**c**) or biologic gel treatment in dark (**e**). While biologic gel in light-treated rats exhibited granulation tissue formation admixed with inflammation (**d**, vertical arrows), the distribution was less widespread, often sparing portions of the dermis (bracket). An intact epidermal layer (asterisks) was more frequently noted in biologic gel in light-treated rats. In contrast, rats that were not treated (**b**) or received gel (**c**) or biologic gel treatment in dark (**e**) exhibited widespread dermal granulation tissue admixed with necrotic tissue and inflammatory cells (horizontal arrows). Note the absence of intact epidermis and ulceration in (**b**,**c**,**e**). The control group received no treatment. Scale bar = 100 µm.

The exemplary wounds at day 7 are shown in Fig. 3a. Significantly smaller wound area and less ulceration were appreciated in wounds that received the novel biologic gel treatment in light compared to wounds that received other treatments. Animals were sacrificed at day 7 for histology evaluations stained with hematoxylin and eosin (H&E). Longitudinal sections of tarsal skins across the wounds demonstrated less extensive ulceration with the biologic gel treatment in light (Fig. 3a). Widespread dermal granulation tissue admixed with necrotic tissue and inflammatory cells with the absence of epidermis was noted in wounds that did not receive the biologic gel treatment in light (Fig. 3b,c,e). In contrast, a less extensive inflammation sparing portion of the dermis with an epidermal layer was observed in wounds that received the biologic gel treatment in light (Fig. 3d).

Quantification of histopathologic findings was also attempted using a grading system. After 7 days of daily treatment, wounds that received the novel biologic gel treatment in light or dark demonstrated the lowest degree of neutrophilic inflammation and tissue necrosis with the highest degree of acanthosis and hyperkeratosis compared to control and HA gel in BG11 treatments (Table S7). The novel biologic gel treatment in light was associated with a similar amount of granulation tissue and mononuclear inflammation compared to control. The biologic gel treatment in dark, in contrast, decreased the amount of granulation tissue and mononuclear inflammation compared to controls.

### Treatment with the novel biologic gel increased tissue oxygenation during acute ischemia with prolonged benefit in the PAD ischemic wound model

**Figure 4:**
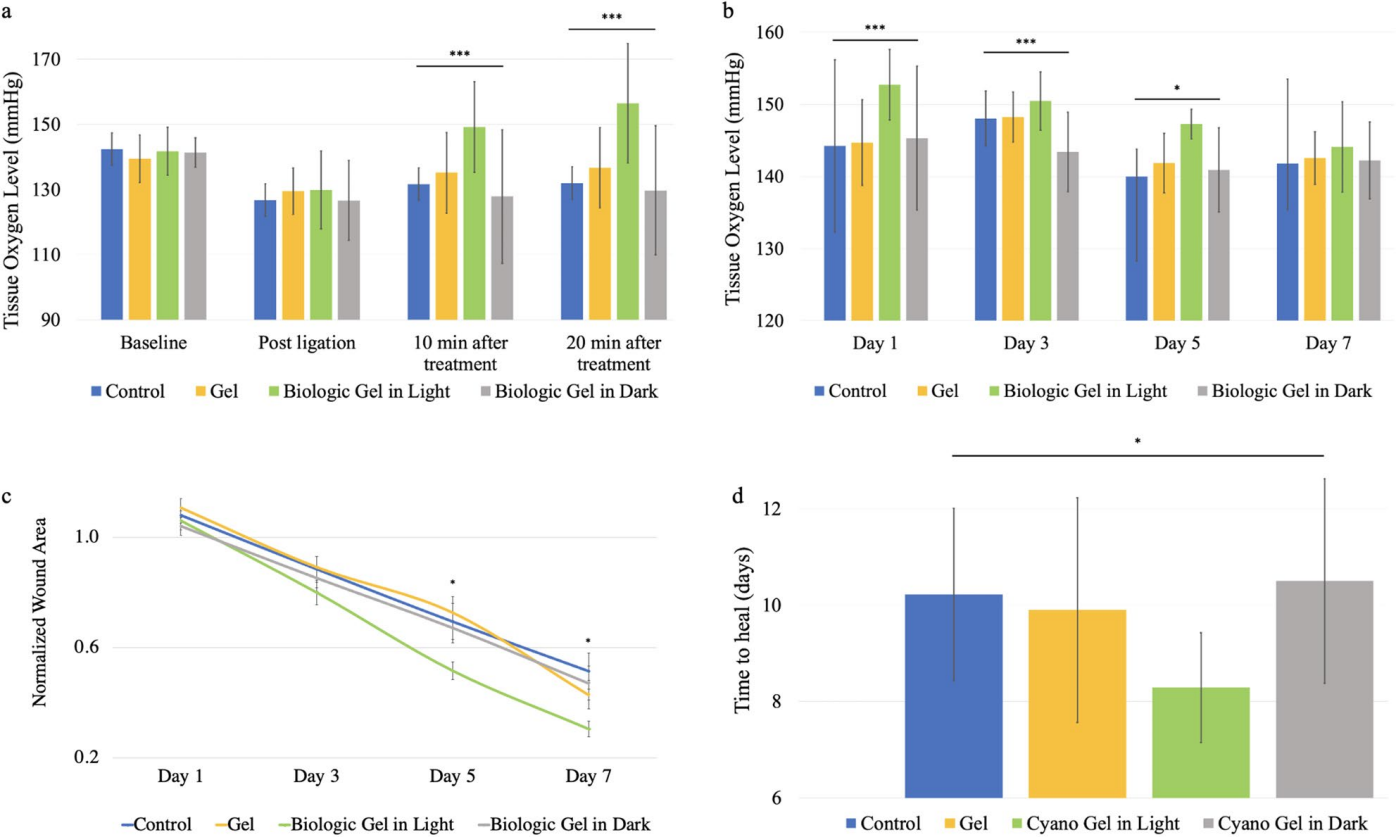
Tissue oxygenation and wound healing speed in a rodent peripheral arterial disease ischemic wound model. (**a**) After ligation, tissue oxygen level decreased from baseline (p < 0.0001 for all groups based on paired t-test). At 10 and 20 min after treatment, tissue oxygen levels were different among the groups at both time points (p < 0.0001) with post-hoc correction illustrating differences between biologic gel treatment in light compared to control, biologic gel treatment in dark, and gel (p = 0.001 for all comparisons at both time points). 40 animals were sacrificed for each treatment group. (**b**) ANOVA analysis demonstrated different tissue oxygenation level on day 1 (p < 0.0001), 3 (p < 0.0001), and 5 (p = 0.039) but not day 7 (p = 0.83) with post-hoc correction showing those that wounds received the novel biologic gel in light treatment demonstrated elevated tissue oxygenation level compared to control, biologic gel treatment in dark, and gel on day 1 (p = 0.001 for all comparisons), compared to biologic gel treatment in dark on day 3 (p = 0.001), and compared to control on day 5 (p = 0.04) after the ischemic injury. For each treatment group, day1: n = 40, day3: n = 32, day5: n = 24, day7: n = 16. (**c**) Normalized wound areas were similar among treatment groups on day 1 (p = 0.52) and day 3 (p = 0.38) but different on day 5 (p = 0.04) and day 7 (p = 0.05) from ANOVA with post-hoc correction showing wounds that received the novel biologic gel in light with the smallest unhealed normalized wound area after 5 days of treatment compared to gel (p = 0.04 from post-hoc correction). For each treatment group, day1: n = 40, day3: n = 32, day5: n = 24, day7: n = 16. (**d**) Wound healing speeds were different among the groups (p = 0.01) with post-hoc correction showing wounds that received the novel biologic gel treatment in light healed the fastest compared to biologic gel in dark treated wounds (p = 0.02) and controls (p = 0.05). Data presented as mean ± standard deviation except for (c) that was presented as mean ± standard error. The controls received no treatment. Controls: n = 9, gel: n = 10, novel biologic gel in light: n = 14, biologic gel in dark: n = 10. Statistics shown on the figure were resulted from ANOVA. * Indicates p < 0.05, ** indicates p < 0.01, and *** indicates p < 0.001.

To eliminate the potential confounding effect of burn injury and to mimic ischemic wounds, a PAD ischemic wound model was generated and studied. Similarly, we first evaluated the effect of the novel biologic gel on tissue oxygenation in vivo by measuring oxygen levels at baseline, 10 min after ischemia, as well as 10 and 20 min after treatment (control without treatment, HA gel in BG11, and novel biologic gel in light or dark) on the distal thigh wounds in the rodent PAD ischemic wound model (Fig. 4a). Baseline tissue oxygen levels were similar among all four groups (p = 0.27) and significantly decreased after the ligation of femoral artery (p < 0.0001 for all groups). The acute ischemic tissue oxygen levels were similar among all four groups prior to treatment (p = 0.51). After 10 and 20 min of treatment, however, animals that received the novel biologic gel treatment in light demonstrated the highest tissue oxygen levels in the wounds compared to those that received other treatments. We then further tracked tissue oxygen level after the acute ischemic injury. Animals that received the novel biologic gel treatment in light demonstrated persistent increased level of tissue oxygenation in the wounds compared to control, biologic gel treatment in dark, and gel on day 1 (p = 0.001 for all 3 comparisons), compared to biologic gel treatment in dark on day 3 (p = 0.001), and compared to control on day 5 (p = 0.04) after the ischemic injury (Fig. 4b). There was no difference in the wound oxygen level 7 days after the ischemic injury among the four treatment groups.

### The novel biologic gel treatment significantly improved wound healing in the PAD ischemic wound model

We evaluated the effect of novel biologic gel on wound healing in the rodent PAD ischemic wound model. We first investigated the wound healing speed by measuring unhealed wound area and then quantifying the time needed for complete wound healing. Wounds that received the novel biologic gel in light demonstrated the smallest unhealed wound area after 5 days of daily treatment compared to wounds that received gel treatment (Fig. 4c). Overall, wounds that received the novel biologic gel treatment in light healed the fastest with an average time to heal of 8.3 ± 1.1 days compared to 10.2 ± 1.8 days without treatment (*p* = 0.05), 9.9 ± 2.3 days with HA gel in BG11 treatment (p = 0.10), and 10.5 ± 2.1 days with biologic gel treatment in dark (p = 0.02, Fig. 4d). No hypertrophic scar was observed in any animals.

**Figure 5:**
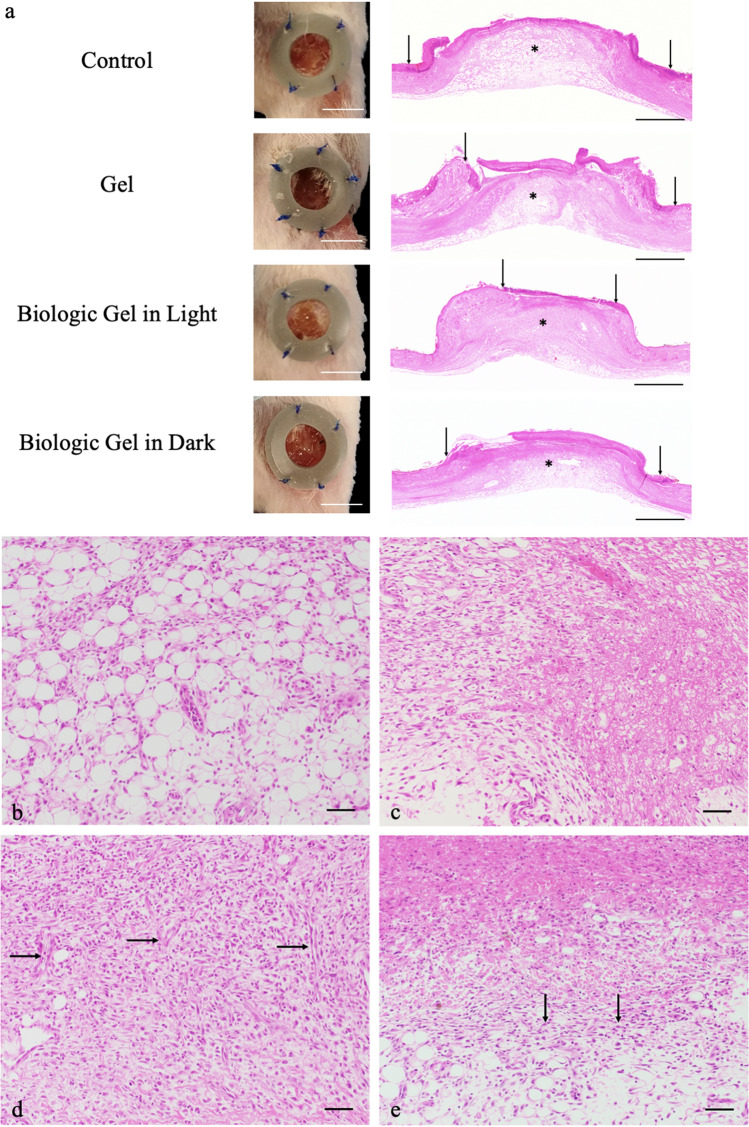
Gross and histopathological evaluation of distal thigh wounds after 3 days of treatment in a rodent peripheral arterial disease ischemic wound model. (**a**) Gross distal thigh lesions after 3 days of daily treatment showed smaller wound area in the wound that received the novel biologic gel treatment in light compared to the wounds that received other treatment. Scale bar = 5 mm. Hematoxylin and eosin stain of longitudinal sections of the distal thigh lesions 3 days after acute ischemic injury showed that wounds that were treated with biologic gel in light generally had increased amounts of dermal and subcutaneous granulation tissue formation (asterisk) compared to those receiving other treatments. Re-epithelialization margins were also generally more central within the wound bed in the wound treated with biologic gel in light compared to those that received other treatments. Vertical arrows denote margins of re-epithelialization. Scale bar = 1 mm. (**b**–**e**) Hematoxylin and eosin stain of cross sections of the distal thigh lesions under higher magnification showed that wounds that were treated with biologic gel in light (**d**) had increased amounts of granulation tissues as evidenced by new blood vessels (horizontal arrows) perpendicularly oriented to fibroblasts and collagen fibers. Granulation tissue was less frequent in controls (**b**, not present), animals treated with gel only (**c**, no present), or animals treated with biologic gel in dark (**e**, vertical arrows). The control group received no treatment. Scale bar = 50 µm.

The exemplary wounds at day 3 are shown in Fig. 5a. Significantly smaller wound area was noted in animals that received the novel biologic gel treatment in light compared to wounds that received other treatments. H&E stained histopathology images on wound biopsies harvested at day 3 are shown in Fig. 5. Longitudinal sections of the distal thigh skins across the wounds demonstrated increased amounts of dermal and subcutaneous granulation tissue formation after novel biologic gel in light treatment compared to those that received other treatments (Fig. 5). Additionally, the re-epithelialization edge generally migrated the most in wounds that were treated with the novel biologic gel in light compared to those that received other treatments (Fig. 5a).

To obtain more granular data, histopathologic evaluations were conducted on 1, 3, 5, 7, and 14 days after treatment. Wound re-epithelialization within the wound bed demonstrated an enhanced trend in animals that received the novel biologic gel in light after 1, 3, and 5 days of treatment compared to those that received other treatments, but this marginal difference disappeared by day 7 (Fig. S5a). By day 14, all animals demonstrated complete re-epithelialization of the wound. Most noticeably, novel biologic gel in light treatment was associated with significantly decreased neutrophilic inflammation in the wounds after 1 and 3 days of treatment compared to the other treatment groups (Fig. S5b). Additionally, animals that received the novel biologic gel in light also demonstrated enhanced early granulation tissue formation and mononuclear inflammation (Fig. S5c,d).

### The novel biologic gel treatment demonstrated anti-inflammatory effect in the PAD ischemic wound model

**Figure 6:**
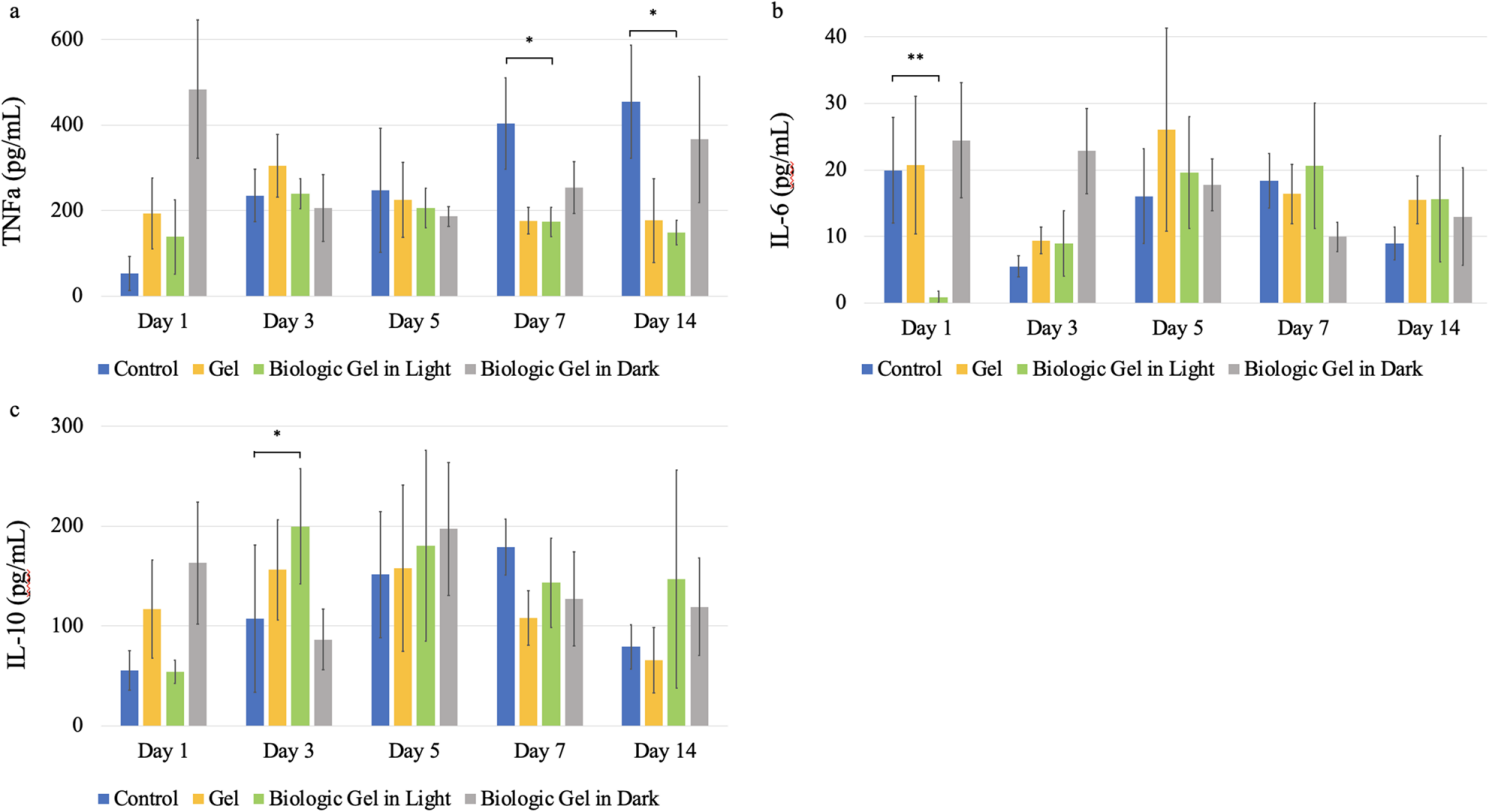
Systemic cytokine effect after treatment in a rodent peripheral arterial disease ischemic wound model. (**a**) Tumor necrosis factor α level in peripheral blood was reduced after 5 days of novel biologic gel in light treatment and was significantly decreased after 7 and 14 days of novel biologic gel in light treatment compared to controls. (**b**) Interleukin 6 level in peripheral blood was significantly reduced after 1 day of novel biologic gel in light treatment compared to controls and remained similar to other treatment groups at the remaining timepoints. (**c**) Interleukin 10 level in peripheral blood was significantly increased after 3 days of novel biologic gel in light treatment compared to controls and remained elevated on day 5 and 14. Data presented as mean ± standard deviation. The control group received no treatment. For each treatment group at each timepoint, 8 animals were sacrificed for measurement. * Indicates p < 0.05 and ** indicates p < 0.01.

To evaluate the systemic cytokine effect after ischemic wounding with the novel biologic gel treatment, enzyme-linked immunosorbent assays (ELISA) were performed to quantify tumor necrosis factor α (TNFα), interleukins 6 (IL-6), and interleukins 10 (IL-10) levels in peripheral blood after 1, 3, 5, 7 and 14 days of treatment (Fig. 6). TNFα levels were lowered on day 5 and significantly reduced after 7 and 14 days of treatment in animals that received the novel biologic gel in light compared to controls (no treatment). IL-6 levels were also found to be significantly reduced in animals that received the novel biologic gel in light treatment compared to controls 1 day after treatment. Additionally, 3 days after novel biologic gel in light treatment was associated with increased IL-10 levels compared to those that did not receive any treatment. There is a trend of persistently elevated IL-10 levels after novel biologic gel in light treatment compared to controls after 5, 7, and 14 days of treatment.

### The novel biologic gel treatment altered local immune cell populations in the PAD ischemic wound model

**Figure 7:**
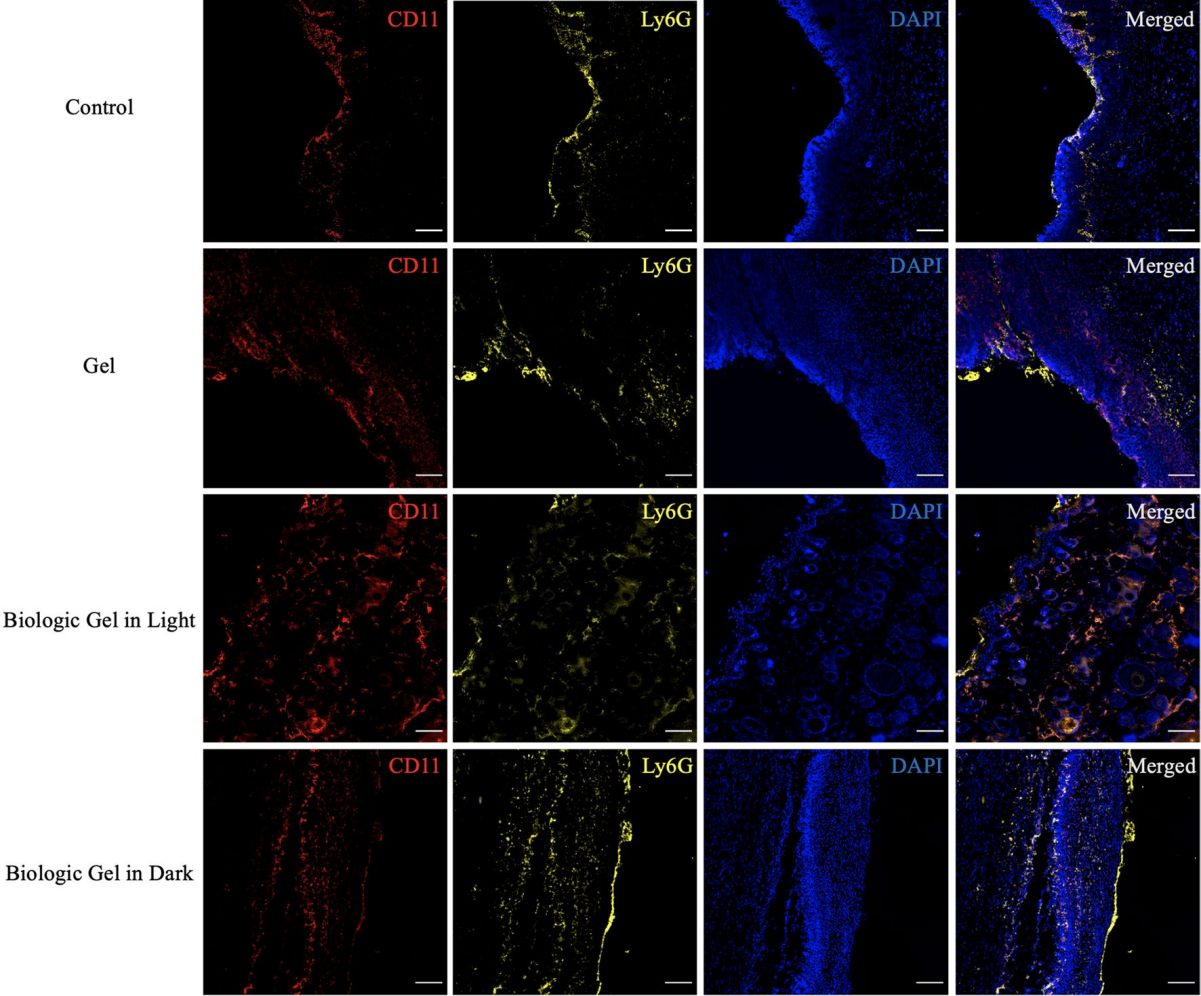
Immunohistochemical staining of local immune cells after 3 days of treatment in a rodent peripheral arterial disease ischemic wound model. Wounds that received the novel biologic gel in light treatment demonstrated the least amount of Lg6G+ cell infiltration in the dermis and subcutaneous tissue compared to wounds that received other treatments. CD11+ cell population remained similar among the four treatment groups. The control group received no treatment. Note that CD3+ split channel images are not shown here as very minimal signals were observed. Red = CD11, yellow = Ly6G, blue = DAPI. Scale bar = 100 µm.

The novel biologic gel treatment was associated with a significant early decrease in Ly6G+ cells compared to controls (no treatment, Fig. S6a). As shown in Fig. 7, exemplary wounds that received the novel biologic gel in light treatment demonstrated less infiltration of Ly6G+ cells in the dermis and subcutaneous tissue after 3 days of treatment compared to wounds that received other treatments. CD11+ cell population appeared to be similar among the four treatment groups during the early wound healing phase (Figs. 7, S6b, S7) until day 14 when wounds that received the novel biologic gel in light treatment showed significantly increased percentage of CD11+ cells compared to controls (Fig. S6b). CD3+ cells remained to be minimal throughout the 14 day treatment period, and the population remained similar among the four treatment groups (Fig. S6c).

### The novel biologic gel treatment preserved the normal skin flora

Baseline skin cultures demonstrated Staphylococcus, Corynebacterium, Enterococcus, Lactobacillus, Streptococcus, and lactose fermenting rods species. No anaerobic bacteria were detected in any of the baseline skin cultures. There were no bacteria identified from samples collected after the skins were prepped prior to surgery and wounding. In terms of aerobic bacteria, both day 1 and day 7 samples after wounding showed normal skin flora, similar to what was identified from baseline cultures. One control animal showed bifidobacterium growth on day 1 after wounding. Otherwise, no anaerobic bacteria were found in other treatment groups. Seven days after treatment, no anaerobic bacteria were identified from the wounds. No cyanobacteria were identified in any wounds at either time points.

### The novel biologic gel was nontoxic with minimal immunogenic effect

Finally, we evaluated the potential in vivo toxicity and immune response to the topical application of our novel biologic gel. Clinically, no animals demonstrated any signs of systemic infection or illness. Animals were sacrificed at day 7 and 14, and peripheral blood was collected and analyzed for complete blood count, C-reactive protein (CRP), and blood culture. A subset of animals underwent comprehensive necropsy evaluation. In both wound models, blood cultures were persistently negative for bacterial growth over 3 weeks for all animals. CRP measured at both day 7 and 14 were 0 mg/L for all animals. Complete blood count results at day 7 and 14 were similar among the four treatment groups in both wound models (Table S8). All animals that underwent necropsy after 7 or 14 days of treatment in either wound models did not exhibit any pathologic findings in any of the tissues or organs examined.

## Discussion

This study demonstrates the novel, elegant translation of *S. elongatus*’s photosynthetic power to restore tissue ischemia from PAD, one of the most common cardiovascular diseases worldwide. Our results showed that *S. elongatus* can be effectively used as a topical agent to considerably accelerate wound healing by augmenting tissue oxygenation, which has profound clinical implications. This topical biologic gel can directly impact the care for patients suffering from chronic limb-threatening ischemia at a high risk of amputation which may only be prevented via invasive revascularization currently^17^.

For the initial design of this innovative therapy, we chose HA as the gel base because it is a naturally occurring polymer within the skin and has been extensively studied for its wound healing properties^18,19^. It has been shown to influence the tissue regeneration process via modulating receptors, inflammatory response, cellular migration, and angiogenesis, all of which are the main phases of wound healing^20–22^. HA is also Food and Drug Administration approved for medical use in a variety of fields from orthopedics to cosmetic surgery^23^. With its important rheostatic and viscosity-controlling properties, this hydrogel creates a locally stable environment to support *S. elongatus* over several weeks with enhanced, sustained oxygen release. It also simplified the dressing application process, further improving the clinical adoptability of this technology. With this novel biologic topical gel treatment, HDFa viability and migration were significantly improved in the hypoxia condition compared to other treatment groups. Note that HDFa migration results assessed via the scratch assay may be affected by cellular proliferation. Interestingly, we did not observe any positive effect from HA gel treatment without *S. elongatus*. We suspect that the differences in HA concentration and molecular weight, which can impact physiologic processes including apoptosis, immunostimulation, and angiogenesis^24,25^, possibly contribute to this observation.

The basis of this innovative therapy is the enhanced tissue bioenergetics from increased tissue oxygen delivery by the *S. elongatus*^26,27^. This principle is heavily used clinically as providers strive to reconstitute blood flow to restore proper tissue oxygenation via a variety of techniques, such as angioplasty and conduit bypass^28^. In our study, we demonstrated a significant increase in tissue oxygen level after the application of our novel biologic gel in light compared to other treatments. Rats are also known to have robust collateral vessels in hindlimbs^29^, which may explain a higher tissue oxygen level measured at the distal thigh compared to in the tarsal area. We also noted a slight increase in oxygen levels in the non-biologic gel in light groups 10 and 20 min after treatment compared to post-ligation measurements. We believe that this is likely secondary to the diffusion of atmospheric oxygen into the wounds.

In this study, we implemented two wound models in an attempt to evaluate the efficacy of the novel biologic gel in a comprehensive manner. We investigated the PAD burn wound model first to imitate acute trauma that could occur to a limb with PAD in a clinical scenario. With the promising results obtained from this first model, a second model, the PAD ischemic wound model, was then studied to eliminate the potential confounding effect of burn injury. This second model was designed such that splints were used to prevent wound healing from contraction^30,31^, thus allowing us to assess wound healing speed and the re-epithelialization process more accurately. However, even though the splint helped to minimize contraction, inevitably there was some degree of wound contraction due to the granulation tissue and fibrosis. Given the open wounds held by the splint, longer-term tissue oxygen levels could be measured, and the novel biologic gel in light treatment was indeed found to have a prolonged effect in tissue oxygenation augmentation beyond the acute period. We also included additional sample harvesting time points to allow for a more granular analysis of the wound healing process after treatment using the novel biologic gel in light.

In addition to tissue oxygenation enhancement, our novel biologic gel in light treatment also appeared to have some effect on improved reparative wound healing with decreased tissue destruction in the PAD burn wound model. The detailed histological examination of the tarsal wound sample revealed a diminished trend in destructive processes, such as slightly decreased neutrophilic inflammation and tissue necrosis, as well as an enhanced trend in reparative activities in animals treated with our novel biologic gel in light. More noticeably, in the PAD ischemic wound model, decreased early neutrophilic inflammation, decreased Ly6G+ cell population, and enhanced early wound healing, evidenced by granulation tissue formation and mononuclear inflammation within the wound bed, were also observed after the novel biologic gel in light treatment. Additionally, re-epithelialization progression was quantified by measuring the epithelial margin from the original wound edge. Enhanced early healing was generally observed again in animals that were treated with novel biologic gel in light compared to other treatments. Note that in most histopathologic markers, the differences among treatment groups in the PAD ischemic wound model disappeared by 7 days after treatment, suggesting that the early phase of wound healing was likely primarily impacted by the novel biologic gel in light treatment. We suspect that this enhanced early wound healing allows for the epithelium to grow over the wound bed. In fact, almost all PAD ischemic wounds were healed by 14 days after treatment. This could also be greatly influenced by the fact that the original wound size from the PAD ischemic wound model was 5 mm in diameter compared to that from the PAD burn wound model, which was 10 mm in diameter. This is also reflected by the longer time needed to heal in general in the PAD burn wound model compared to the PAD ischemic wound model.

Another interesting observation was the anti-inflammatory effect of the novel biologic gel in light treatment. Specifically, systemic TNFα level was shown to be significantly reduced after biologic gel in light treatment compared to control. In fact, raised local and systemic levels of TNFα were observed in animal models of impaired wound healing^32,33^. By inhibiting TNFα, studies showed that leukocyte recruitment and NFκB activation could be blunted, altering the balance between M1 and M2 macrophages, and therefore accelerating wound healing^34^. Additionally, increased systemic IL-10 levels were also observed, especially during the early phase of wound healing, after the treatment of novel biologic gel in light compared to controls. IL-10 is known as a potent anti-inflammatory cytokine. Several studies have shown its ability to facilitate regenerative healing through regulation of the inflammatory response and as a regulator of the extracellular matrix, fibroblast cellular function, and endothelial progenitor cells^35,36^. The treatment of topical novel biologic gel in light associating with these systemic cytokine level alterations is fascinating and may be an interesting area for future investigations.

Our group has previously demonstrated the safety of *S. elongatus-*based therapy when administered systemically^12,37^. Across all metrics, animals that received *S. elongatus* responded comparably to control animals, displaying no clinically significant immune response. For topical application, we hypothesized that physiologic responses to *S. elongatus*, if any, would be further minimized. As expected, we did not observe any adverse response associated with topical *S. elongatus* treatment. Other systemic inflammatory and infection markers such as neutrophil count, lymphocyte count, platelet count, and CRP levels were comparable among the four treatment groups in both wound models.

Since S. elongatus has been shown to have antibacterial activity^38^, we attempted to ascertain whether the novel biologic gel treatment could alter the microbial flora within the wound bed. Chronic ischemic wounds exhibit a stark perturbation of the normal skin microbial flora^39^. Interestingly, we did not observe any new microorganism in the wound microenvironment. No cyanobacteria were seen in the wound bed, which could be due to the self-grooming behavior of rodents and possible ingestion of the biologic gel. Furthermore, 1 out of 5 control animals showed bifidobacterium growth 1 day after wounding, whereas the novel topical biologic gel treatment was not associated with anaerobic bacterial growth at any time point. Perhaps the biologic gel plays a role in preventing changes in microbes, but a more comprehensive study is needed to fully address this question.

One limitation of this study was the inability to provide prolonged, targeted light exposure in vivo. In order to maximize the effect size, animals would have to be placed in light for the entire duration of the study, which would severely affect their immune response and hormonal responses^40–42^, and inevitably impact the results from this study. A comparison between ligated and unligated animals should also be done to further ensure that the ischemic wounds heal more slowly compared to non-ischemic wounds. Although an increased trend in re-epithelialization was observed after the novel biologic gel in light treatment compared to other treatments, the difference was not statistically significant. We suspect that the novel biologic gel treatment enhanced wound healing within the wound bed, which allowed for epithelium to grow over the wound, rather than directly cause an increase in epithelium migration. Furthermore, it has been shown that rodent wounds heal by contraction whereas human wounds close by granulation tissue^43^. The effect of this novel therapy on epithelialization may not be fully understood. Future experiments should be performed to assess this novel topical gel’s effect on human epidermal keratinocytes in terms of viability and migration in the hypoxic condition. The next important step of this study would involve pre-clinical large animal model validation followed by human clinical trials, where we will be able to provide high-intensity light exposure to the wound area by using a targeted light source. This can be easily translated to direct patient care. A localized, portable light source such as the biliblanket, a phototherapy device to treat neonatal jaundice, could be simply wrapped around the affected limb covering the wound, delivering targeted light exposure to ensure maximal efficiency of the novel biologic gel. Another potential example includes fiber-optic-containing materials in a sock form to further enhance adoptability of this technology. Without interfering with patients’ lifestyle or daily activities, tissue oxygenation can be effectively restored with this novel treatment to accelerate healing and perhaps further prevent ischemic wound in PAD.

In summary, we designed a novel, highly effective, yet simplistic therapeutic approach in treating ischemic wounds associated with PAD by harnessing the photosynthetic power of *S. elongatus*. This topical therapy can be easily applied to other wound etiologies that may benefit from increased oxygen delivery to the injured tissue. Given the low cost of *S. elongatus* propagation, the ease of clinical translation of this technology, and the profound wound healing effect revealed in this study, this novel therapy can provide a highly effective, affordable avenue in treating this challenging patient population. Our novel photosynthetic biologic topical gel shows tremendous potential to generate a paradigm shift in treating ischemic wounds associated with PAD and can potentially influence the clinical care for millions of patients suffering from PAD.

## Methods

### Experimental design

The objectives of this study were to characterize and optimize the biologic gel composition with *S. elongatus *in vitro and to evaluate the in vivo wound healing response of two rodent wound models after being treated with the topical *S. elongatus* biologic gel. For the in vitro testing, three gel compositions with BG11 medium, sterile normal saline, and sterile mili-Q water and three *S. elongatus* concentrations at 30, 60, and 100 million cells per mL were tested for both cell viability and oxygen production with (n = 10) and without light (n = 6). To control for the possible additional effect of hyaluronic acid (HA) on *S. elongatus* survival and oxygen production, the same three solutions without HA that had equal sample numbers were analyzed and compared. The novel biologic gel composition that was associated with the highest *S. elongatus* viability and oxygen level was selected as the final composition for the remainder of the studies. In vitro validation of the efficacy of this biologic gel was carried out using human dermal fibroblasts (HDFa) for viability and migration assessment via scratch assay under hypoxia condition. In addition to the treatment group with the novel biologic gel in light, the HDFa samples were also treated with PBS, HA gel, and *S. elongatus* in PBS as controls. Ten samples were used for each treatment group for the viability study, 5 samples for each treatment were used for the scratch assay.

For the in vivo experiment, two rodent wound models were created and tested. In the first model, PAD was introduced by surgically ligating the right femoral artery, after which, a 1 cm circular tarsal wound was induced by using a low temperature cautery surgical pen. In this model, four treatment groups were created, and animals were randomly assigned to: control without treatment (n = 12), treatment with the novel biologic gel in light (n = 13), biologic gel in dark (n = 10), and HA gel (n = 10). Four control animals, 6 in the biologic gel in light group, 4 in the HA gel group, and 4 in the biologic gel in dark group were sacrificed 7 days after treatment. Four animals in the control and biologic gel in light group, and 3 in the other two groups were sacrificed 14 days after treatment. Wound tissues were explanted for histology assessment. One animal from each group sacrificed on day 7 and 14 underwent necropsy. Peripheral blood was collected for each animal and analyzed for immune response and culture. To assess wound healing time, the animals were sacrificed at day 14. A wound was considered healed when there was complete re-epithelialization and wound closure without discharge, drainage, or scab^44^. All other animals were sacrificed at the previously assigned euthanization timepoint. Additional in vivo experiments were replicated to generate 6 animals per treatment group to confirm the wound healing time.

To eliminate the potential confounding effect of burn injury and to mimic ischemic wounds, a second wound model was generated. This model was also used to obtain longitudinal tissue oxygen level and to quantify immune cells and cytokines that are important to wound healing. PAD was introduced in the same fashion as described above. Next, full thickness dermal excision of 5 mm in diameter was performed on the ipsilateral distal thigh, followed by splint fixation to prevent wound contraction. Animals were randomly assigned to the same four treatment groups (n = 40 per group). In each treatment group, animals were sacrificed at 1, 3, 5, 7, and 14 days (n = 8 per group per time point) after wound introduction and treatment. All animals had wound tissues explanted for histology assessment. Three animals from each treatment group sacrificed on day 7 and 14 underwent necropsy. Peripheral blood was collected for a subset of animals sacrificed on day 7 and 14 and analyzed for immune response and culture. All samples were evaluated by an independent veterinary pathologist. Throughout the study, the individuals who collected results, the operating surgeon, and the veterinary pathologist were blinded to the intervention until the data was ready for analysis.

Lastly, to assess wound microorganism changes after the application of topical treatment, the PAD ischemic wound model was used, and 20 animals were randomized to receive 4 different treatments (n = 5 per group) and received daily treatment and dressing changes as described above. Aerobic, anaerobic, and cyanobacteria cultures were obtained at baseline, after surgical sterile preparation, day 1 and day 7 after treatment. All animals were sacrificed on day 7 after the last wound cultures were collected.

### *Synechococcus elongatus* culture and propagation

*Synechococcus elongatus* slant cultures (catalog no. PCC 7942, Institut Pasteur, Paris, France) were procured from the Pasteur Culture Collection, aliquoted, and frozen in BG11 medium (catalog no. A1379901, Gibco, Carlsbad, CA, USA) with 5% v/v of DMSO at − 80 °C until use. Frozen *S. elongatus* aliquots were thawed rapidly in a 37 °C water bath, centrifuged at 3000×*g* for 5 min at room temperature and resuspended into a 25 mL sterile glass Erlenmeyer flask containing 10 mL of warm BG11 (catalog no. A1379901, Gibco, Carlsbad, CA, USA). The cultures were incubated for 48 h in a Thermo Forma Orbital Shaker (Thermo Fisher Scientific, Model 420, Waltham, MA, USA) at 125 rpm in the dark at 34 °C. Two 15W aquarium fluorescent bulbs (Part Number 22920, General Electric, Boston, MA, USA) were placed on the incubator to provide light exposure to the culture. The culture was maintained by adding fresh, warm BG11 medium every other day and based on optical density measurements to replace evaporative losses and maintain a constant volume. Every 4 days, or when the colony becomes noticeably oversaturated, as indicated by clumping or sediment collecting at the bottom of the flask, the optical density of the stocks were measured using Spectronic Genesys 6 (Thermo Electron Corporation Instruments LLC, Madison, WI, USA) at a wavelength of 750 nm, and cultures were diluted with fresh BG11 media. Throughout the study, purity checks were performed regularly either by ensuring no growth when the liquid cultures were plated on Luria broth agar, or by ensuring no heterogeneous bacterial subpopulations by microscopy.

### Biologic gel preparation

HA gel was prepared by mixing 2% w/w of HA powder of 1 megadalton (catalog no. 024477, Lifecore Biomedical, Chaska, MN, USA) with BG11 medium, sterile normal saline, or sterile mili-Q water in a 50 mL Falcon sterile polypropylene conical tube (catalog no. 14-432-22, Thermo Fisher Scientific, Waltham, MA, USA). The tube was inverted several times to initiate a thorough mixture of the powder and left at room temperature for up to 48 h for the gel to reach a less viscous state. The gel was then sterilized under UV light for 1 h and was stored at room temperature until future use.

To prepare the novel biologic gel with *S. elongatus*, cyanobacteria concentration was calculated using Eq. (1), where C is calculated concentration and OD is optical density measured at a wavelength of 750 nm.

1 $$C=\left(2\times {10}^{8}\right)\times OD-\left(2\times {10}^{6}\right) \;\;\text{cells}/\text{mL}$$

The respective volume of *S. elongatus* was centrifuged at 3000*g* for 5 min at room temperature and resuspended in warm BG11 medium. The appropriate concentration of *S. elongatus* was inoculated in the HA gel in 5 mL glass tubes. The glass tube openings were covered with Parafilm M wrapping film (catalog no. S37440, Thermo Fisher Scientific, Waltham, MA, USA) for the biologic gel with light, and covered fully with aluminum for the biologic gel without light. Both biologic gel tubes had ventilating holes created on top for aeration and were incubated at 34 °C in the orbital shaker for 24 h prior to use. On alternate days, the biologic gels were mixed manually with a sterile metal spatula for thorough aeration. For the in vivo study, fresh biologic gel using BG11 medium at a concentration of 100 million *S. elongatus* cells/mL was made weekly.

### *Synechococcus elongatus* viability study

The viability of *S. elongatus* was assessed in BG11 medium, sterile normal saline, sterile mili-Q water, and in HA gel made from BG11 medium, sterile normal saline, and sterile mili-Q water at a concentration of 30 million cells per mL. The viability of *S. elongatus* was also assessed in HA gel made from BG11 medium, sterile normal saline, and sterile mili-Q water at concentrations of 30 million, 60 million, and 100 million cells per mL. After *S. elongatus* inoculation, the samples were incubated at 34 °C in the orbital shaker. At 24 and 48 h, the samples were transferred to sterile 2.0 mL Axygen microcentrifuge tube and stained using the live/dead dye solution from the LIVE/DEAD *Bac*Light Bacterial Viability kit (catalog no. L7007, Invitrogen, Carlsbad, CA, USA) as directed by the instructions. The samples were vortexed and spun down gently using the mini centrifuge. The samples were wrapped in aluminum foil and incubated for 1 h at room temperature. After the incubation period, the tubes were vortexed gently and 200 µL of 4% Paraformaldehyde solution (catalog no. AAJ1994, Thermo Fisher Scientific, Waltham, MA, USA) was added and the tubes were incubated at room temperature for 20 min for fixation. The LEICA DMi8 microscope (Leica Microsystems, Buffalo Grove, IL, USA) was used at 10× magnification to visualize *S. elongatus*. The fluorescent images were acquired using the LAS X Life Science Microscope Software (Leica Microsystems, Buffalo Grove, IL, USA) and analyzed using ImageJ (NIH, Bethesda, MD, USA).

### Cell culture maintenance

HDFa were purchased commercially (catalog no. C0135C, ThermoFisher Scientific, Carlsbad, CA, USA) and cryopreserved as primary cultures. They were cultured in DMEM (catalog no. 11885076, Gibco, Carlsbad, CA, USA) which was supplemented with 10% HyClone Fetal Bovine Serum (catalog no. SH30071.01, GE Healthcare Life Sciences, Malborough, MA, USA), and 1% Penicillin–Streptomycin (catalog no. 15140122, Gibco, Carlsbad, CA, USA). The culture medium was changed every other day. Growth was monitored using the EVOS XL Core Cell Imaging System (catalog no. AMEX1100, Thermo Fisher Scientific, Waltham, MA, USA).

### Sample preparation and hypoxia treatment for human dermal fibroblasts viability study

When HDFa cells were confluent, they were treated with Trypsin (catalog no. 25200056, Gibco, Carlsbad, CA, USA), lifted, and transferred to a 50 mL Falcon sterile polypropylene conical tube. The cells were centrifuged at 3000*g* for 5 min at room temperature, and the supernatant were removed. Trypan Blue (catalog no. 15250061, Gibco, Carlsbad, CA, USA) was used, and the cells were enumerated on Countess II (catalog no. AMQAF1000, ThermoFisher, Waltham, MA, USA). 3 × 10^5^ HDFa cells were seeded on each well of the Corning Costar 24 Well Cell Culture plate (catalog no. 3524, Corning, Kennebunk, ME, USA). Cells were incubated at 37 °C and 5% CO_2_ for 24 h. Next, culture media were aspirated and were replaced with serum-free DMEM, which was composed of the basal DMEM medium (catalog no. A1443001, Gibco, Carlsbad, CA, USA), 1% l-Glutamine (catalog no. 25030081, Gibco, Carlsbad, CA, USA), 1% Sodium Pyruvate (catalog no. 11360070, Gibco, Carlsbad, CA, USA), and 1% Penicillin–Streptomycin. HDFa cells were serum starved for 24 h at 37 °C and 5% CO_2_ for 24 h. The cells were then placed in hypoxia condition using the Xvivo System at 37 °C and 2% O_2_ (BioSpherix, Model X3, Parish, NY, USA) for 4 h prior to treatment. The control group received 250 µL of PBS (catalog no. 10010049, Gibco, Carlsbad, CA, USA), and the other control groups received 250 µL of HA gel in BG11 or 250 µL of *S. elongatus* solution in PBS at a concentration of 100 million *S. elongatus* cells/mL. The treatment group received 250 µL of the novel biologic gel at a concentration of 100 million *S. elongatus* cells/mL incubated in light. All HDFa cells were retrieved after 24 h of incubation at 37 °C and 2% O_2_ in light. HDFa cells after hypoxia treatment were stained using the LIVE/DEAD Viability/Cytotoxicity kit for mammalian cells (catalog no. L3224, Invitrogen, Carlsbad, CA, USA) as directed by the instructions. Cells were imaged using the fluorescence microscopy (Keyence BZ-X810, Keyence Corp, Osaka, Japan) at 20× magnification using the GFP (catalog no. OP-87763, Osaka, Japan) and mCH/TR (catalog no. OP-87765, Osaka, Japan) filter cubes. The images were then analyzed using ImageJ.

### Sample preparation and hypoxia treatment for human dermal fibroblasts scratch assay

HDFa cells were lifted and counted in the same fashion as described above. 1.5 × 10^5^ cells were plated on a Corning Falcon Polystyrene 12 well plate (0877250, Thermo Scientific, Waltham, MA, USA). Cells were incubated at 37 °C and 5% CO_2_ for 24 h. Next, cells were placed in hypoxia conditions using the Xvivo System at 37 °C and 2% O_2_ (BioSpherix, Model X3, Parish, NY, USA) for 4 h prior to treatment. Then, a scratch wound was generated in each plate by using a sterile pipette tip. Images of each well were taken at 10× magnification using the EVOS XL Core Cell Imaging System to obtain the baseline wound area. Treatment was then immediately added. The control group received 250 µL of PBS, and the other control groups received 250 µL of HA gel in BG11 or 250 µL of *S. elongatus* solution in PBS at a concentration of 100 million *S. elongatus* cells/mL. The treatment group received 250 µL of the novel biologic gel at a concentration of 100 million *S. elongatus* cells/mL incubated in light. All HDFa cells were retrieved after 6, 18, and 24 h of incubation at 37 °C and 2% O_2_ in light for serial wound area assessment using the EVOS XL Core Cell Imaging System. Images were then analyzed using ImageJ to quantify wound areas. Normalized wound area was calculated by dividing the measured wound area at each timepoint by the baseline measurement at time 0 immediately after injury.

### Animal care and biosafety

Male Wistar rats (300 to 350 g) were obtained from Charles River. Food and water were provided ad libitum. All animals were handled in accordance with the Guide for the Care and Use of Laboratory Animals published by the US National Institutes of Health (publication No. 85-23, revised 1996). The experimental protocol (28921) was approved by the Institutional Animal Care and Use Committee at Stanford University, which is accredited by the Association for the Assessment and Accreditation of Laboratory Animal Care. The authors complied with the ARRIVE guidelines.

### Rodent peripheral artery disease wound models

The well-established rodent hindlimb ischemia model was used to generate the peripheral arterial disease model^45,46^. Briefly, rats were anesthetized using 2–3% inhaled isoflurane (Fluriso, VetOne, Boise, ID, USA) delivered at 1 L/min. Anesthesia during mechanical ventilation was maintained using continuous 1–2% isoflurane via nose cones (Harvard Apparatus VentElite, Holliston, MA, USA). The right femoral artery was exposed through a 2-cm skin incision made immediately below the femoral ligament. The femoral artery was dissected free from the surrounding tissue. Prior to ligation, a small stab incision was made on the dorsal surface of the ipsilateral foot or on the external surface of the distal thigh on the ipsilateral side for the PAD burn wound model or the ischemic wound model, respectively. Tissue oxygen level was measured through the small stab incision. Next, using a 4-0 prolene suture, the femoral artery and its branches were ligated. The femoral incision was then closed in layers. Ten minutes after femoral artery ligation, tissue oxygen level was measured again. For the PAD burn wound model, a low temperature cautery surgical fine tip pen of 1 mm in diameter and a maximal temperature of 704 °C (Bovie Medical Corporation, Clearwater, FL, USA) was used to create a circular lesion of 1-cm in diameter. This was achieved by first marking the wound edge on the tarsal skin. Then, the cautery surgical pen was directly placed to the skin in a circular motion from the perimeter to the center for 1 min in total. This allowed for a consistent depth of injury to the skin without damage to the underlying tissues. For the PAD ischemic wound model, a 5 mm dermal punch biopsy (RoyalTek Company LTD, Taoyuan City, Taiwan) was used to generate a full thickness wound on the distal thigh as previously described^43^. Next, wound splinting was performed, which has also been previously published^30,31^. In this study, a 3D-printed wound splint (SIL 30, Carbon, Redwood City, CA) with an inner diameter of 5 mm and a thickness of 2 mm was placed on top of the punch wound so that the inner opening space of the splint lined up with the punch wound. 5-0 polypropylene sutures were used to secure the splint onto the adjacent skin in an interrupted fashion. The animals were randomized, and the wounds received HA gel in BG11, the novel biologic gel in light, biologic gel in dark or no treatment (control). Tissue oxygen levels were measured at 10 and 20 min after treatment. For animals that received biologic gel in dark, the experiments were carried out with dimmed room lights. Immediately after the application of biologic gel in dark, the wounds were covered with aluminum foil to shield the wounds from any ambient light exposure. Animals’ wounds that received biologic gel in dark were dressed in gauze, aluminum foil, followed by tegaderm to prevent light exposure. All other animals’ wounds were dressed in gauze and tegaderm to allow normal light exposure. Animals were then recovered from anesthesia. All animals were housed in our regular rodent husbandry rooms with ambient light exposures for 12 h a day. The treatments and dressings were reapplied every day, and the unhealed wound areas were measured on 1, 3, 5, and 7 days after surgery. Normalized wound area was calculated by dividing the measured wound area at each timepoint by the baseline measurement at day 0 immediately after injury.

Complete wound healing was defined as full epithelialization without discharge, drainage, or scab.

### Oxygen level measurement

Oxygen level was measured both in vitro and in vivo using the OxyLite Pro XL O2 Sensor (Oxford Optronix, Abingdon, England, United Kingdom) and the OxyLite NX pO_2_ bare fiber sensor probe (Oxford Optronix, Abingdon, England, United Kingdom). Ambient oxygen level was measured prior to each sample measurement. An oxygen level greater than 200 mmHg was registered as 200 mmHg, the maximal measurable oxygen level using this sensor.

### Terminal blood collection and processing

For the PAD burn wound model, animals were sacrificed 7 or 14 days after treatment. For the PAD ischemic wound model, animals were sacrificed 1, 3, 5, 7, or 14 days after treatment. This was achieved by humanely euthanizing the animals via CO_2_ asphyxiation. The haired skin overlying the chest was shaved and sterilized with 70% ethanol. A clam shell thoracotomy was performed to expose the heart. 10 mL of whole blood was drawn from the heart. For animals that were sacrificed on day 7 or 14, 2 mL of whole blood was collected in sterile Signal Blood culture bottles (Thermo Scientific) for microbiological culture using the Biolog system, and another 2 mL of whole blood was collected into dipotassium EDTA‐coated BD Microcontainer tubes for complete blood counts and C-reactive protein. The remaining blood were collected in 15 mL falcon tubes (14-959-53A, Corning Inc., Corning, NY) without anticoagulant and left undisturbed at room temperature for 30–60 min for ELISA. Animals sacrificed on all other days had all 10 mL of whole blood stored in the 15 mL falcon tubes for ELISA.

### ELISA assay and analysis

TNFα (TNFα, ELR-TNFα-1; RayBiotech Life Inc., Peachtree Corners, GA), IL-6 (ELR-IL-6-1, RayBiotech Life Inc., Peachtree Corners, GA), and IL-10 (ELR-IL-10-1, RayBiotech Life Inc., Peachtree Corners, GA) ELISA kit reagents and serum samples were thawed and cooled to room temperature before use. The ELISAs were performed according to the kit instructions. A microplate reader (7131000, BioTek Instruments Inc., Winooski, VT) and Gen 5 software (5321001, BioTek Instruments Inc., Winooski, VT) were used to measure the absorbance values of the standards and samples at a wavelength of 450 nm. The standards and samples were analyzed in duplicates, and their absorbance values were averaged. The absorbance value of the blank was subtracted from the absorbance values of the standards and samples. Using GraphPad Prism 9.0.0 software for Windows (GraphPad, San Diego, CA), a nonlinear regression was fitted to the standard concentration vs standard absorbance data points on a logarithmic scale. The line-of-best fit equation was used to calculate concentration values from the sample absorbance measurements.

### Wound biopsy, necropsy, and histopathology

Gross images of the wounds were obtained from all animals. For the PAD burn wound model, rats underwent right tarsal biopsy following euthanasia. An approximately 1-cm-wide strip of haired skin was excised from the level of the right distal tibia to the phalanges. For the PAD ischemic wound mode, regions of haired skin containing experimental wounds were removed from the right distal thigh and undermined along fascial planes. The location of the splint was inked with Margin Marker (Vector Surgical™, Waukesha, WI, USA) to measure the extent of re-epithelialization. The excised haired skin containing the wounds were longitudinally bisected. One hemisection was laid flat in the optimum cutting temperature compound (OCT, Fisher HealthCare) using 2-methylbutane on dry ice. The samples were stored at − 80 °C until use. The second hemisection was placed in a tissue cassette to retain tissue orientation and immersion-fixed in 10% neutral buffered formalin for 72 h.

In addition to tarsal and thigh biopsy, a subset of rats underwent gross necropsy. Routine tissues including heart, lung, liver, spleen, kidney, adrenal gland, eyes, testes, accessory sex glands, salivary gland, pancreas, lymph node, tongue, trachea, thyroid gland, thymus, esophagus, stomach, small intestine, large intestine, skin, brain, white and brown adipose tissue were immersion-fixed in 10% neutral buffered formalin for 72 h. Formalin-fixed tissues were then processed routinely, embedded in paraffin, sectioned at 5 µm, and stained with hematoxylin and eosin (H&E). Photomicrographs of H&E sections were taken on an Olympus BX60 microscope (Olympus, Shinjuku City, Tokyo, Japan) equipped with an Olympus DP27 camera using Olympus cellSens imaging software. All tissues were blindly evaluated by a board-certified veterinary pathologist for tarsal and thigh wound healing and evidence of off-target tissue toxicity.

### Histopathology scoring

Ordinal histopathologic grading scales were designed to evaluate for tarsal and thigh wound healing. Briefly, tissues were scored from 0 to 4 based on neutrophilic inflammation, granulation tissue, mononuclear inflammation, tissue necrosis (tarsal wounds only), and acanthosis and hyperkeratosis (tarsal wounds only) with 0 being normal, 1 being ≤ 25% tissue affected, 2 being 25–50% tissue affected, 3 being 50–75% tissue affected, and 4 being ≥ 75% tissue affected. For the thigh wound study, re-epithelialization was quantified by measuring the distance from the inner margins of the tissue ink which marks the initial wound edge to the inner leading edges of re-epithelialization from both sides of a sectioned sample. The distance was then averaged for each sample for further analysis.

### Immunohistochemical staining

The hemisection samples from the PAD ischemic wound model stored in OCT underwent cryosection at 10-μm in the cross-sectional direction. The sectioned samples were rinsed with PBS, incubated with 4% PFA at room temperature, then in 0.5% PBS-Tween, and finally incubated with 10% FBS for 60 min in room temperature. PBS was used to rinse the samples between each step. The sections were then incubated with CD11b/c mouse anti-rat antibody, clone: OX-42 (catalog no. MA190756, Invitrogen, dilution 1:100), anti-CD3 monoclonal antibody (catalog no. MA1-90582, Thermo Fisher Scientific, dilution: 1:500), and anti-Ly6G antibody (catalog no. GTX40912, GeneTex, dilution: 5 µg/ml) at 37 °C for 90 min. The slides were then incubated in the dark for 45 min at 37 °C using secondary antibodies with a 1:200 dilution. The secondary antibodies consisted of goat anti-rabbit IgG H&L (Alexa Fluor 488 ab150077-500ug, Abcam), donkey anti-rat IgG H&L (Alexa Fluor 647 ab150155-500ug, Abcam), and goat anti-mouse IgG H&L (Alexa Fluor 594 ab150116-500ug), respectively. Finally, the sections were incubated with DAPI (catalog no. R37606, NucBlue Fixed Cell ReadyProbes Reagent, ThermoFisher Scientific). The stained slides were kept at 4 °C until imaging.

### Immunohistochemical fluorescent image acquisition and analysis

After immunohistochemical staining, the samples were imaged by segments using the fluorescence microscopy (Keyence BZ-X810, Keyence Corp, Osaka, Japan) at 20X magnification using the GFP (catalog no. OP-87763, Osaka, Japan), mCH/TR (catalog no. OP-87765, Osaka, Japan), Cy5 (catalog no. OP-87766, Osaka, Japan), and DAPI (catalog no. OP-87762, Osaka, Japan) filter cubes. The images were then stitched together using the Keyence BZ-X800 Analyzer to generate complete wound images. Using ImageJ, three 400 × 400 µm areas including the dermal and subcutaneous tissues were randomly selected. For each selected area, the total number of cells was determined by manually counting the positively DAPI stained cells. For each antibody stain, the number of positively stained cells were determined by manually counting cells that displayed both DAPI and the corresponding antibody signal. For each sample, the cell counts for each antibody stain and the total number of cells were calculated by taking the sum of each measurement from the 3 selected areas. The percentage of cells stained positively for each antibody was then calculated. All images were captured and analyzed in a blinded fashion until the final data was ready for analysis.

### Wound microorganism analysis

To assess wound microorganism changes after the application of topical treatment, the PAD ischemic wound model was used. Twenty animals were randomized to receive 4 different treatments (n = 5 per group) and received the corresponding treatments with daily dressing changes. Specifically, baseline cultures were first obtained prior to any manipulation to the skin where the ischemic wounds would be. After sterile preparation of the surgical field using alternating betadine and ethanol wipes, wound cultures were obtained to confirm proper sterilization. Day 1 and day 7 wound cultures were then obtained prior to daily treatment application and dressing changes. Betadine was used to clean just the outside of the wound prior to sample collection. Aerobic, anaerobic, and cyanobacteria cultures were collected using Eswabs and sterile swabs at all time points. All animals were sacrificed on day 7 after the last wound culture collection.

To inoculate, incubate, and analyze the wound culture, Eswabs were streaked in a semi-quantitative fashion onto pre-reduced Brucella agar, blood agar, chocolate agar, columbia nalidixic acid agar, and MacConkey agar. The sterile swabs were submerged in BG11 media. For aerobic cultures, they were incubated at 35 °C for 5 days and read daily. Anaerobic cultures were placed in an anaerobic container with a gas generator and an anaerobic indication strip. These were then incubated in anaerobic conditions for 48 h at 35 °C and read daily, followed by 3 additional days for re-incubation. Identification of the microorganism was performed by a microbiologist in a blinded fashion based on colony morphology, hemolytic reaction, colony color on differential media, gram stain, wet mount, catalase, and pyrrolidonyl-β-naphthylamide. Isolation was performed by picking a single, well-isolated colony and streaking it to the appropriate non-selective media. For anaerobic cultures, they were streaked to pre-reduced brucella agar. For aerobic cultures, they were streaked to blood agar. Anaerobes were ruled out or ruled in by using an aero-tolerance test to determine the nature of the organism, followed by gram stain and identifications using the Remel RapID Rana kit.

### Statistical analysis

Statistical analyses were performed using SAS version 9.4 (SAS Institute Inc., NC, USA). Continuous variables were reported as mean ± standard deviation unless specified otherwise. Sample variance was assessed using F-test. Comparison between and among treatment groups were performed using student t-test and analysis of variance (ANOVA), respectively. If ANOVA was significant, Turkey post-hoc correction was performed to assess individual group treatment effect. A p-value of less than 0.05 was considered statistically significant.

### Ethics declarations

All animals were handled in accordance with the Guide for the Care and Use of Laboratory Animals published by the US National Institutes of Health (publication No. 85-23, revised 1996). The experimental protocol (28921) was approved by the Institutional Animal Care and Use Committee at Stanford University, which is accredited by the Association for the Assessment and Accreditation of Laboratory Animal Care. The authors complied with the ARRIVE guidelines.

## Supplementary Information

Supplementary Information.

## Data Availability

Data will be made available upon reasonable request to the corresponding author.

## Abbreviations

CRP: C-reactive protein
HA: Hyaluronic acid
HDFa: Adult human dermal fibroblast
H&E: Hematoxylin and eosin
PAD: Peripheral artery disease
