# Supplementary material for: A novel photosynthetic biologic topical gel for enhanced localized hyperoxygenation augments wound healing in peripheral artery disease
Source: Sci Rep. 2022 Jun 15;12:10028. doi: 10.1038/s41598-022-14085-1 (PMC9200759; doi:10.1038/s41598-022-14085-1)
Supplement: Supplementary file 1 — Supplementary Information. [file 41598_2022_14085_MOESM1_ESM.docx]

**SUPPLEMENTARY MATERIALS**

**A Novel Photosynthetic Biologic Topical Gel for Enhanced Localized Hyperoxygenation Augments Wound Healing in Peripheral Artery Disease**

Yuanjia Zhu, MD, MS^1,2^, Jinsuh Jung, BS^1^, Shreya Anilkumar, BA^1^, Sidarth Ethiraj, MS^1^, Sarah Madira, BS^1^, Nicholas A. Tran, BS^1^, Danielle M. Mullis, BS^1^, Kerriann M. Casey, DVM, DACVP^3^, Sabrina K. Walsh^1^, Charles J. Stark, BS, MS^1^, Akshay Venkatesh, BS^1^, Alexander Boakye^1^, Hanjay Wang, MD^1^, Y. Joseph Woo, MD^1,2^

^1^ Department of Cardiothoracic Surgery

^2^ Department of Bioengineering

^3^ Department of Comparative Medicine

Stanford University, Stanford, CA

**
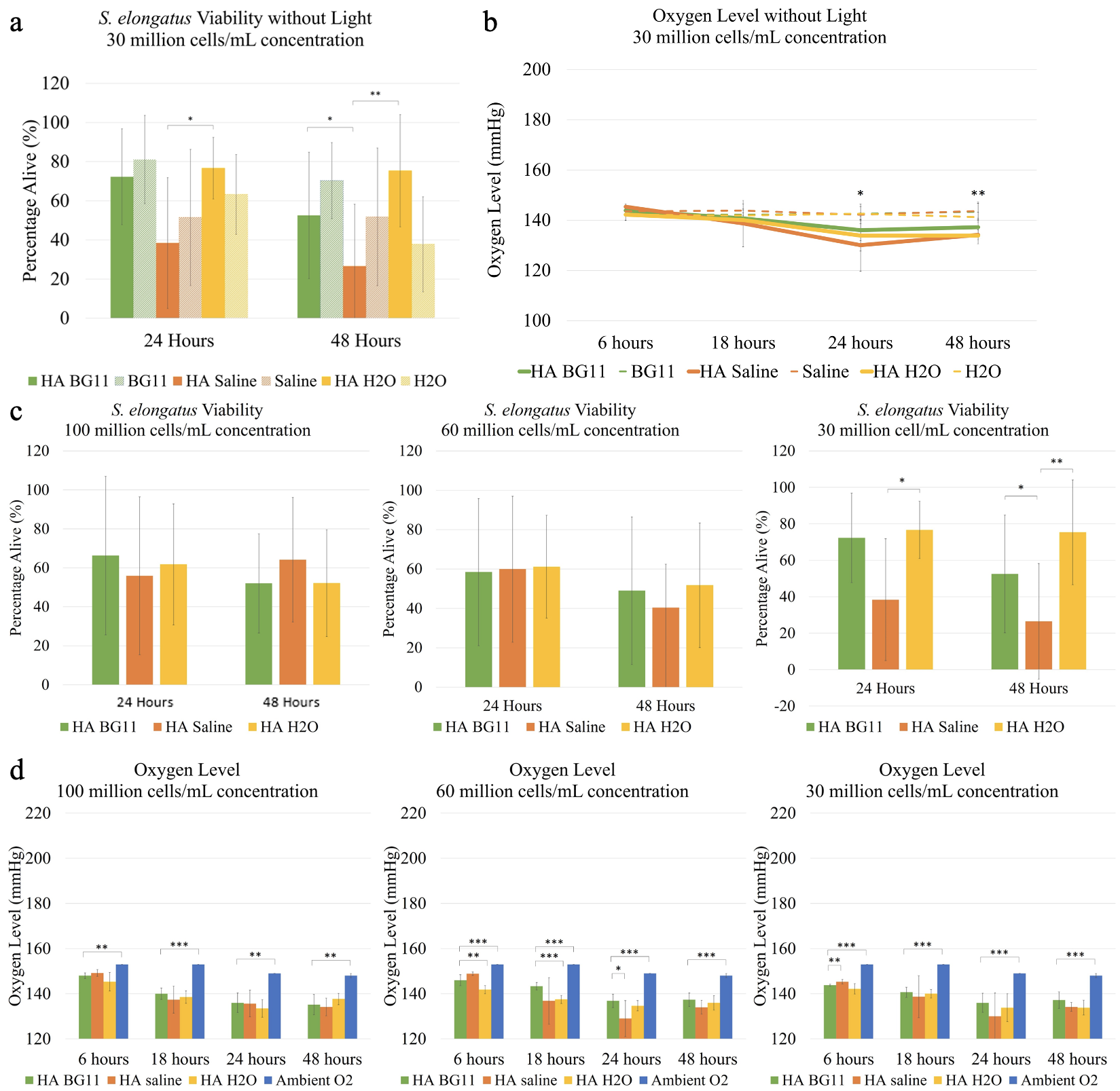
**

**Fig. S1: Effect of hyaluronic acid on *S. elongatus* viability and oxygen production without light exposure.** (**a**) *S. elongatus* viability at 30 million cells/mL in BG11 medium, normal saline, and H_2_O with and without hyaluronic acid were similar between the groups and after 24 compared to 48 hours of incubation. Statistical analyses were performed using the student t test. (**b**) At 30 million cells/mL, the addition of hyaluronic acid to BG11 medium, normal saline, and H_2_O was associated with lower level of oxygen at 24 and 48 hours. The addition of hyaluronic acid to BG11, normal saline, and water was associated with higher oxygen level at 24 and 48 hours (p < 0.007, p < 0.02, and p < 0.01, respectively, based on post-hoc correction). Statistical analyses shown in the figure were performed using the analysis of variance at each time point. (**c**) *S. elongatus* viability at 30, 60, or 100 million cells/mL concentration remained similar within the same gel composition at 24 and 48 hours. Statistical analyses were performed using the student t test. (**d**) Oxygen level in all gel composition at 30, 60, or 100 million cells/mL concentration were lower than ambient oxygen level without light exposure. Statistical analyses shown in the figure were performed using the student t test at each time point. Data presented as mean ± standard deviation. A total of 6 samples were tested for each gel composition for each treatment at each time point. * Indicates p < 0.05, ** indicates p < 0.01, and *** indicates p < 0.001.

**
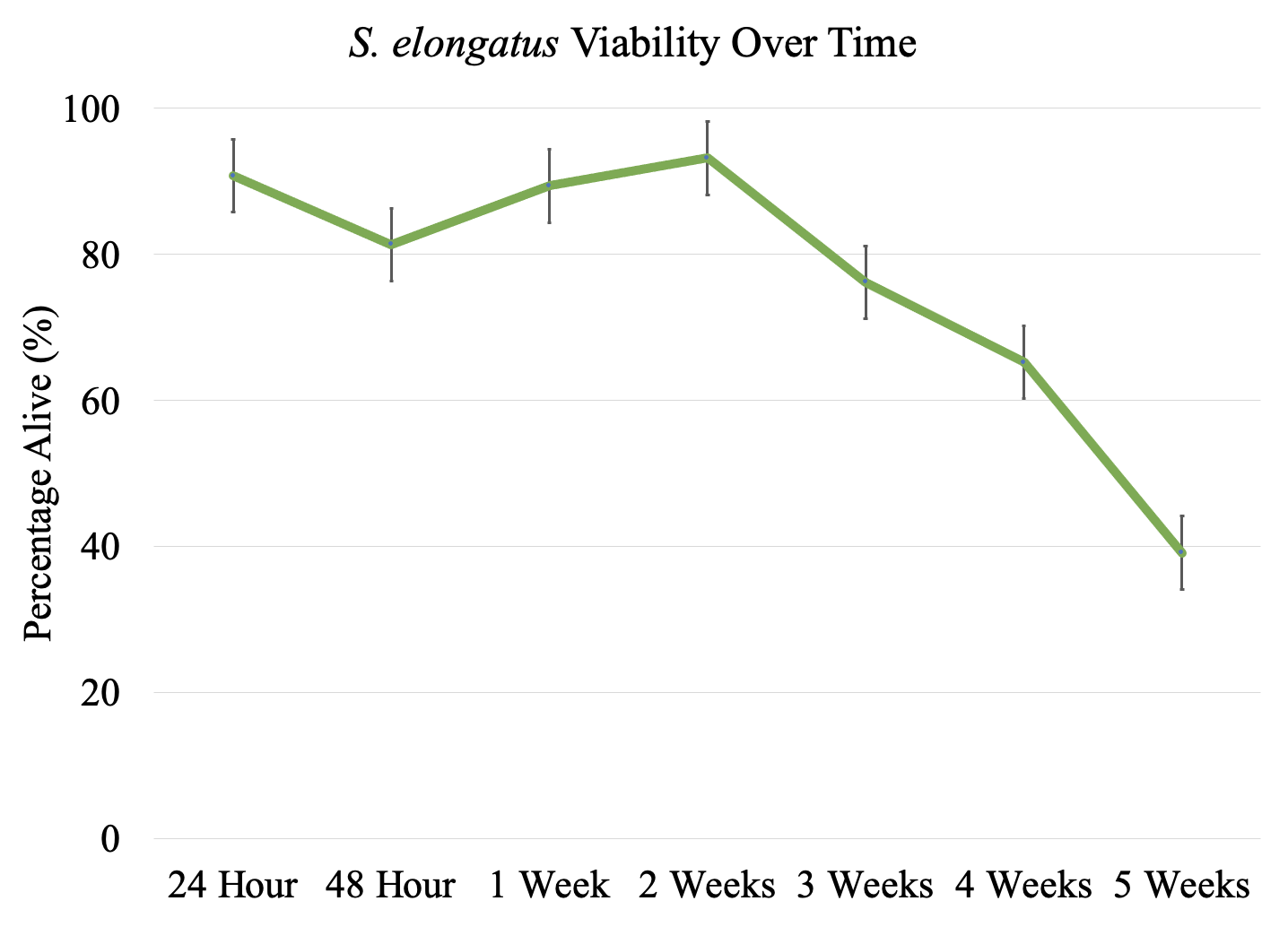
**

**Fig. S2: *S. elongatus* viability over 5 weeks**. The novel biologic gel supported high *S. elongatus* viability of over 80% for 2 weeks. Data presented as mean ± standard deviation. A total of 5 samples were measured at each time point.


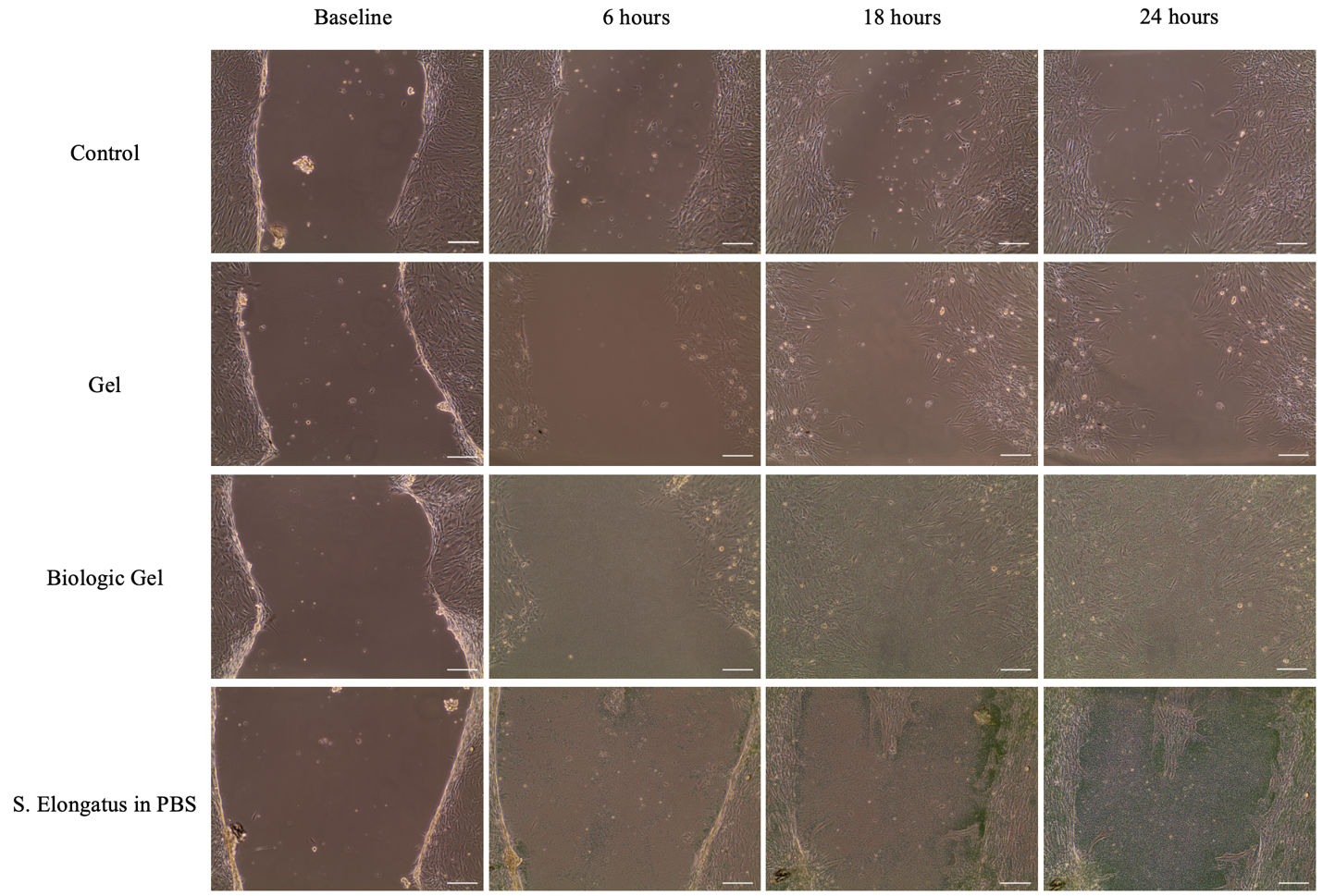


**Fig.S3: Exemplary images of human dermal fibroblast scratch assay experiments.** The biologic gel treatment was associated with the fastest wound healing compared to all other treatments. Scale bar = 200 µm.


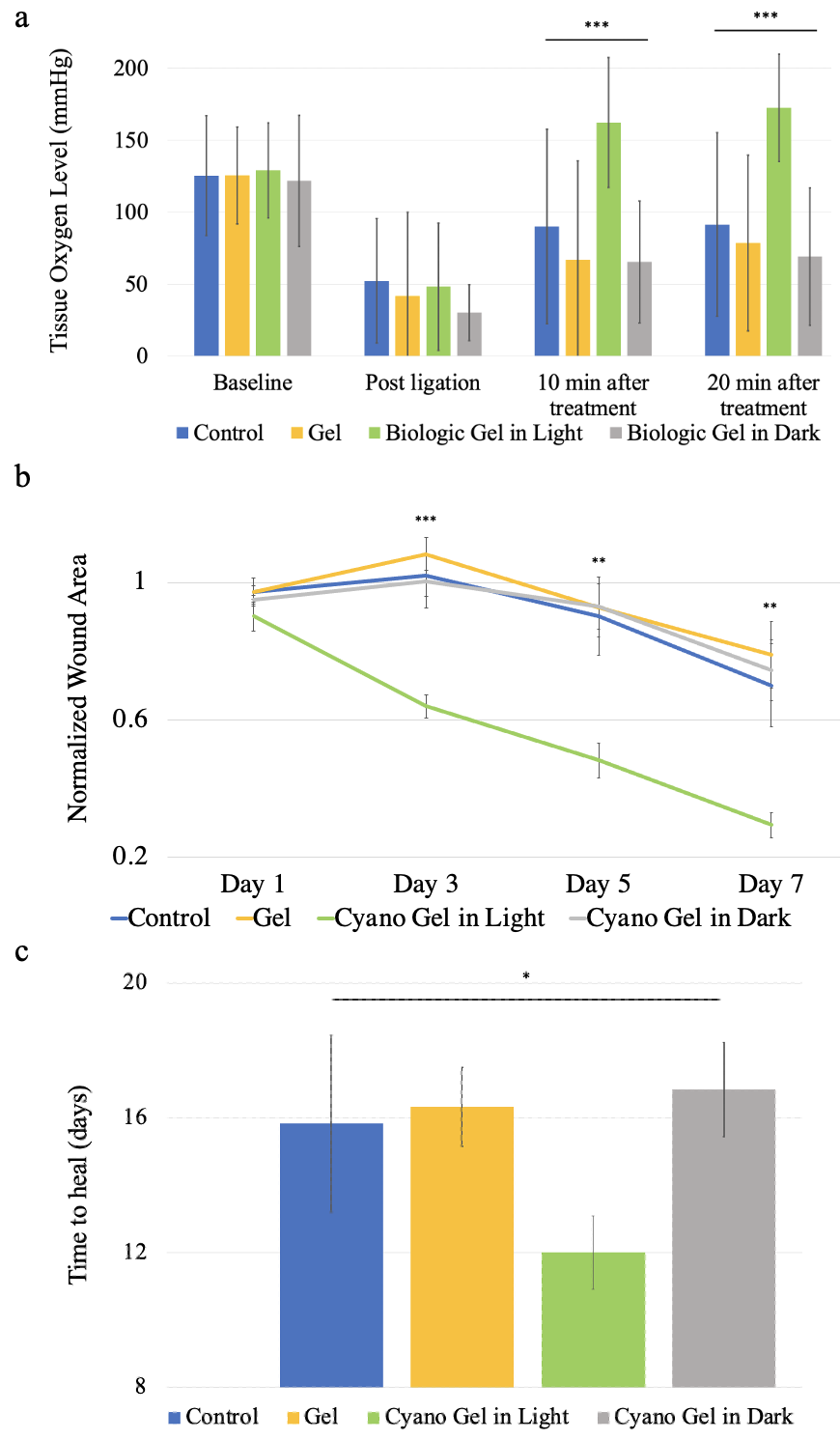


**Fig. S4: Tissue oxygenation and wound healing speed in a rodent peripheral arterial disease burn wound model**. (**a**) After femoral arterial ligation, ipsilateral tarsal tissue oxygen level significantly decreased compared to that measured at baseline prior to femoral arterial ligation. The biologic gel treatment in light significantly increased tissue oxygen level at 10 and 20 minutes compared to that of control wounds (p = 0.004 and p = 0.0007, respectively, based on post-hoc correction). Control without treatment: n = 12, gel: n = 10, novel biologic gel in light: n = 13, biologic gel in dark: n = 10. Data presented as mean ± standard deviation. (**b**) Wounds that received the novel biologic gel in light demonstrated the smallest unhealed normalized wound area after 3, 5, and 7 days of daily treatment compared to control wounds (p = 0.0004, p = 0.02, and p = 0.03, respectively, based on post-hoc correction). Control without treatment: n = 12, gel: n = 10, novel biologic gel in light: n = 13, biologic gel in dark: n = 10. Data presented as mean ± standard error. (**c**) Wounds that received the novel biologic gel treatment in light healed the fastest compared to control wounds (p = 0.03, based on post-hoc correction). N = 6 for each treatment group were measured. Data presented as mean ± standard deviation. Statistics shown on the figure were all resulted from the analysis of variance. The control group received no treatment. * Indicates *p* < 0.05, ** indicates *p* < 0.01, and *** indicates *p* < 0.001.


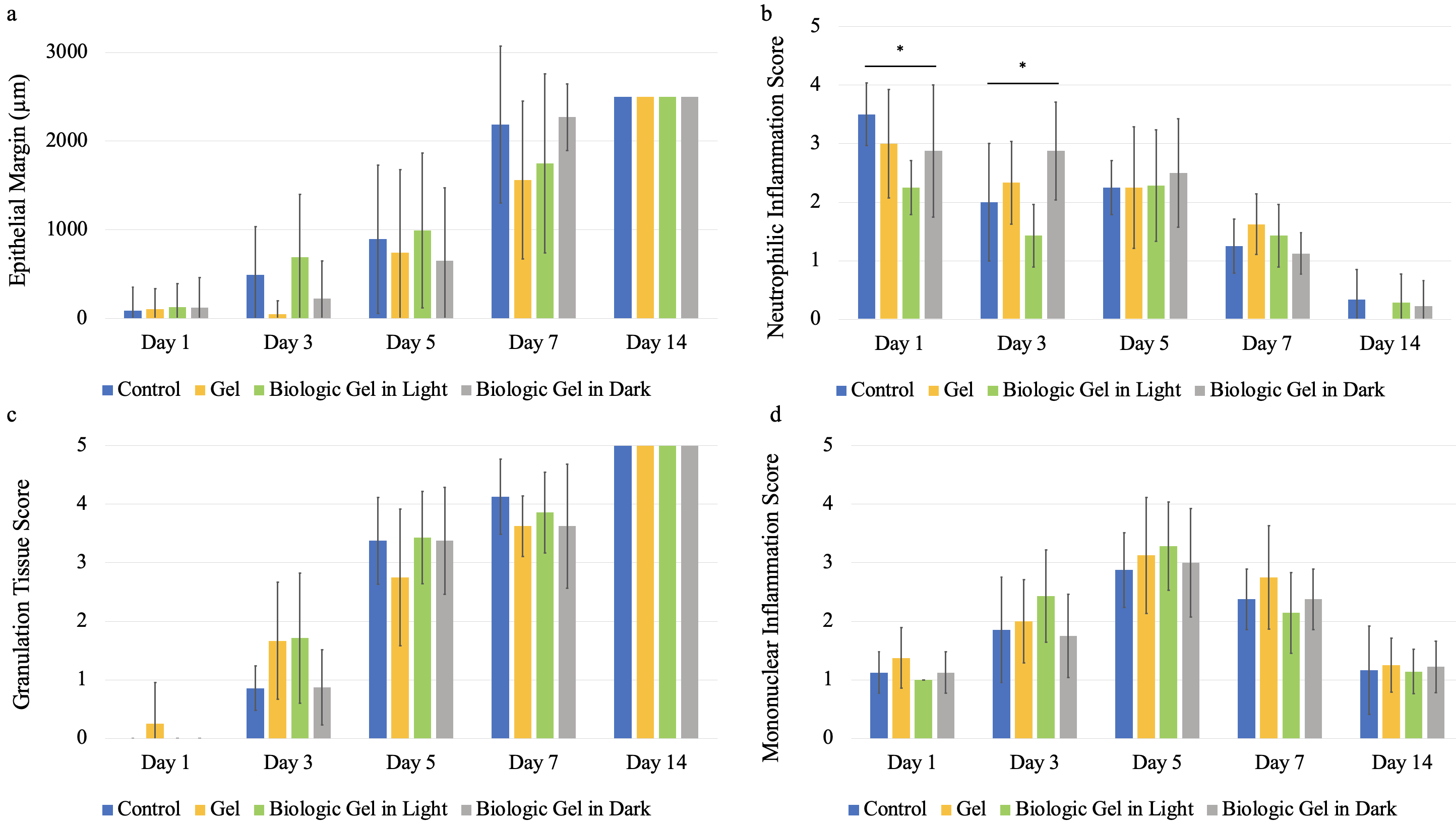


**Fig. S5: Wound histology quantification and mean scoring from a rodent peripheral arterial disease ischemic wound model.** (**a**) Epithelialization margin measured from the original wound edge demonstrated qualitatively enhanced healing in animals that received the novel biologic gel in light after 1, 3, and 5 days of treatment compared to those that received other treatments. (**b**) Novel biologic gel in light treatment was associated with significantly decreased early neutrophilic inflammation in the wounds compared to controls at day 1 (p = 0.0002 based on post-hoc correction). (**c**) Increased early granulation tissue formation at day 1 and day 3 was observed in wounds that received novel biologic gel in light treatment compared to other treatments. (**d**) Increased early mononuclear inflammation from day 1 to day 5 was noted in wounds that received novel biologic gel in light treatment compared to other treatments. For each treatment group, day 1: n = 40, day 3: n = 32, day 5: n = 24, day 7: n = 16, day 14: n = 8. Data presented as mean ± standard deviation. Statistical analyses shown in the figure were all performed using the analysis of variance. The control group received no treatment. * Indicates *p* < 0.05.


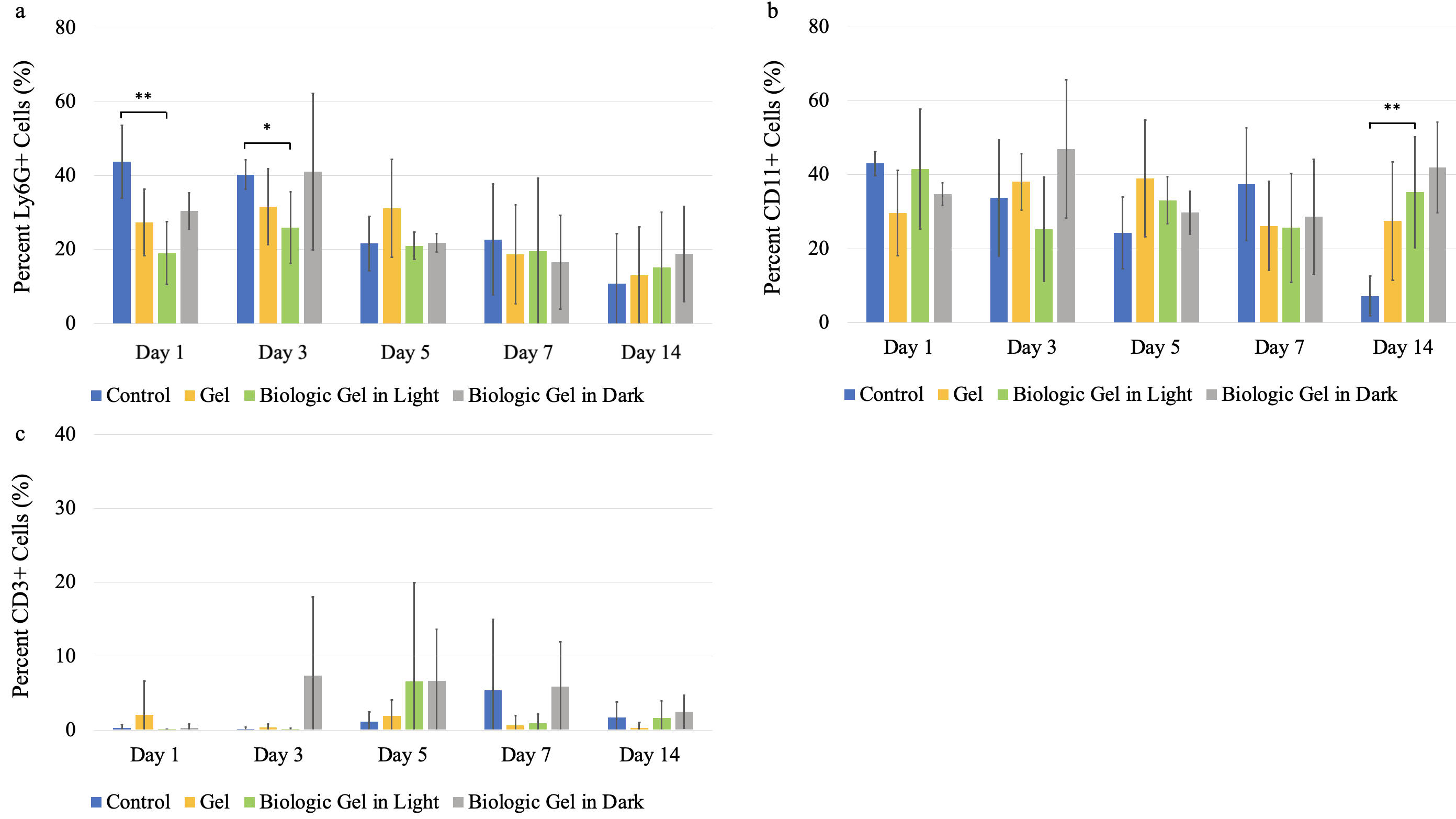


**Fig. S6: Quantification of local immune cells in a rodent peripheral arterial disease ischemic wound model.** (**a**) Wounds that received the novel biologic gel in light treatment demonstrated decreased percentages of Ly6G+ cells after 1 and 3 days of treatment compared to wounds that received no treatment (controls). (**b**) CD11+ cell percentages remained similar among the four treatment groups until day 14 where wounds that received the novel biologic gel in light treatment demonstrated increased percentages compared to wounds that received no treatment. (**c**) CD3+ cell percentages remained similar and low among the four treatment groups throughout. For each treatment group at each timepoint, 5 animals were sacrificed for measurements. Data presented as mean ± standard deviation. Statistical analyses shown in the figure were all performed using the student t test. The control group received no treatment. * Indicates *p* < 0.05 and ** indicates *p* < 0.01.


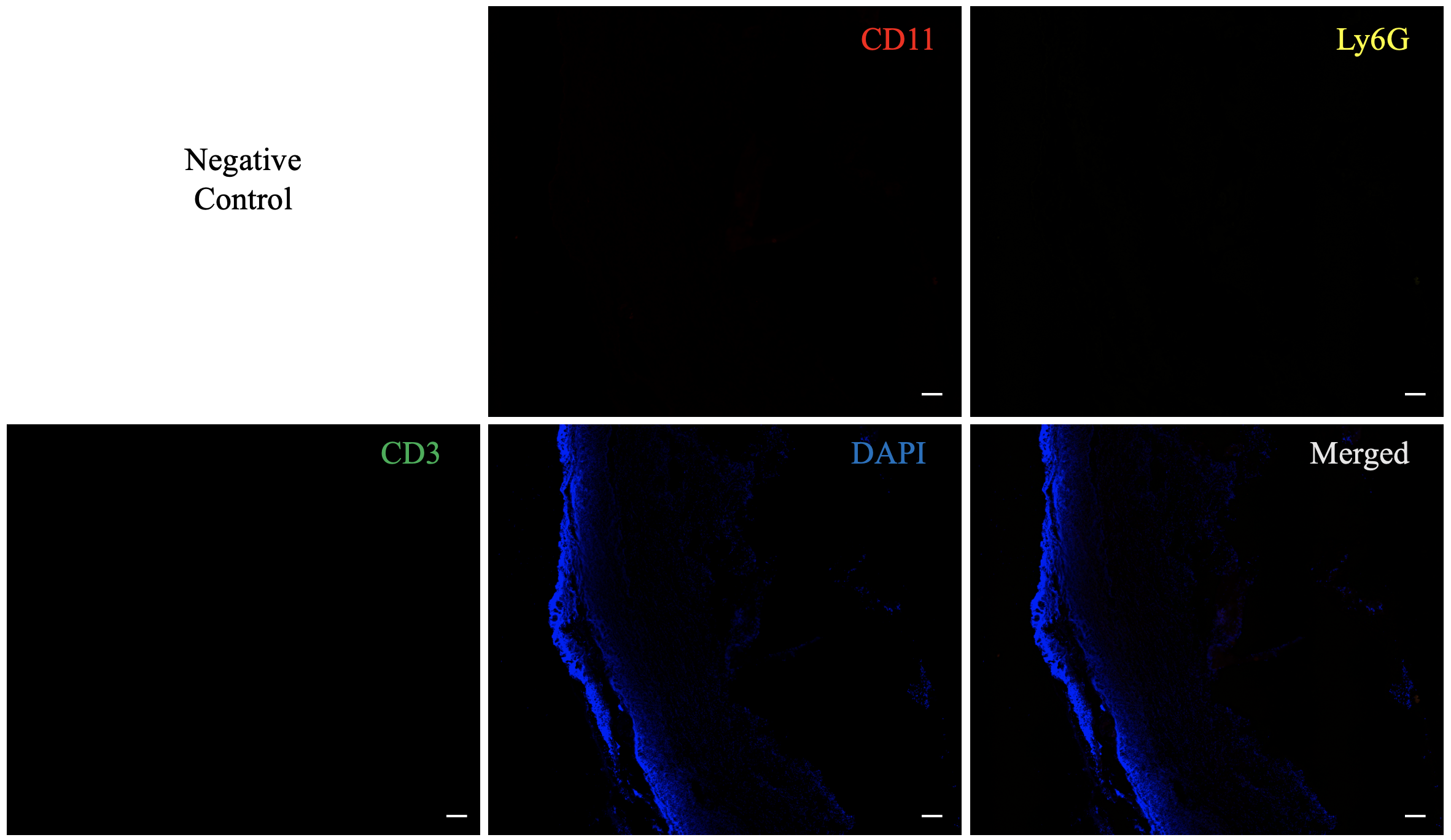


**Fig. S7: Negative control of immunohistochemical staining.** Wound that received no treatment for 3 days after the injury was stained using the secondary antibodies without the primary antibodies for CD11, Ly6G, or CD3. Note minimal to absence of signal in split channel images for CD11, Ly6G, and CD3. Red = CD11, yellow = Ly6G, green = CD3, blue = DAPI. Scale bar = 100 µm.

**Table S1: Effect of hyaluronic acid on *S. elongatus* viability with and without light exposure after 24 and 48 hours of incubation.**

| **Viability (%)** | **HA BG11**  **(n = 10)** | **BG11**  **(n = 10)** | **P value** | **HA Saline**  **(n = 10)** | **Saline**  **(n = 10)** | **P value** | **HA H_2_O**  **(n = 10)** | **H_2_O**  **(n = 10)** | **P value** |
| --- | --- | --- | --- | --- | --- | --- | --- | --- | --- |
| Light 24 hours | 85.4±13.3 | 79.9±15 | 0.83 | 67.4±24.0 | 73.8±16.8 | 0.77 | 67.1±33.5 | 76.5±27.4 | 0.26 |
| 48 hours | 91.3±13.2 | 90.2±7.3 | 0.83 | 74.6±31.4 | 77.7±29.1 | 0.82 | 78.0±21.2 | 74.1±30.7 | 0.75 |
| **Viability (%)** | **HA BG11**  **(n = 6)** | **BG11**  **(n = 6)** | **P value** | **HA Saline**  **(n = 6)** | **Saline**  **(n = 6)** | **P value** | **HA H_2_O**  **(n = 6)** | **H_2_O**  **(n = 6)** | **P value** |
| Dark 24 hours | 72.3±24.5 | 81.1±22.6 | 0.32 | 38.4±33.5 | 51.5±34.8 | 0.48 | 76.7±15.7 | 63.3±20.4 | 0.42 |
| 48 hours | 52.5±32.3 | 70.3±19.4 | 0.58 | 26.5±31.7 | 51.8±35.2 | 0.12 | 75.4±28.7 | 37.8±24.3 | 0.33 |

HA, hyaluronic acid.

Data presented as mean ± standard deviation. P value compared each solution base with and without HA. Statistical analyses performed using the student t test.

**Table S2: Effect of hyaluronic acid on *S. elongatus* oxygen production with and without light exposure.**

| **O_2_ level (mmHg)** | **HA BG11**  **(n = 10)** | **BG11**  **(n = 10)** | **P value** | **HA Saline**  **(n = 10)** | **Saline**  **(n = 10)** | **P value** | **HA H_2_O**  **(n = 10)** | **H_2_O**  **(n = 10)** | **P value** |
| --- | --- | --- | --- | --- | --- | --- | --- | --- | --- |
| Light 6 hours | 156.4±9.8 | 147.3±1.5 | 0.01 | 146.5±3.1 | 145.7±3.5 | 0.6 | 144.6±3.7 | 146.6±3.4 | 0.22 |
| 18 hours  24 hours  48 hours | 173.5±13.6  183.7±16.5  187.2±13.6 | 154.1±2.5  150.2±3.9  152.7±3.4 | 0.0003  <0.0001  <0.0001 | 143.2±3.5  137.0±4.0  138.7±4.0 | 147.8±2.9  146.8±3.3  145.4±2.8 | 0.005  <0.0001  0.0004 | 138.3±5.1  135.1±6.3  137.6±4.5 | 145.8±4.2  145.4±4.3  146.9±4.8 | 0.002  0.0005  0.0007 |
| **O_2_ level (mmHg)** | **HA BG11**  **(n = 6)** | **BG11**  **(n = 6)** | **P value** | **HA Saline**  **(n = 6)** | **Saline**  **(n = 6)** | **P value** | **HA H_2_O**  **(n = 6)** | **H_2_O**  **(n = 6)** | **P value** |
| Dark 6 hours | 143.8±0.4 | 142.3±1.8 | 0.07 | 145.3±1.0 | 144.3±2.2 | 0.33 | 142.2±2.3 | 142.2±2.2 | 1 |
| 18 hours  24 hours  48 hours | 140.7±2.3  136.0±4.2  137.2±3.7 | 142.2±2.6  142.5±2.3  143.3±1.6 | 0.31  0.008  0.004 | 138.7±9.2  130.0±10.3  134.2±1.9 | 143.8±2.6  142.2±0.8  143.7±3.5 | 0.22  0.02  0.0002 | 140.0±1.9  133.8±6.1  133.8±3.3 | 141.8±2.2  142.5±2.9  141.7±2.3 | 0.16  0.01  0.0007 |

HA, hyaluronic acid.

Data presented as mean ± standard deviation. P value compared each solution base with and without HA. Statistical analyses performed using the student t test.

**Table S3: Effect of *S. elongatus* concentration on its viability.**

| **Viability (%)** | **HA BG11**  **(n = 10)** | **HA Saline**  **(n = 10)** | **HA H_2_O**  **(n = 10)** | **P value** | **HA BG11**  **(n = 10)** | **HA Saline**  **(n = 10)** | **HA H_2_O**  **(n = 10)** | **P value** |
| --- | --- | --- | --- | --- | --- | --- | --- | --- |
|  | **24 hours** | | | | **48 hours** | | | |
| Light 30 million/mL | 85.4±13.3 | 67.4±24.0 | 67.1±33.5 | 0.08 | 91.3±13.2 | 74.6±31.4 | 78±21.2 | 0.17 |
| 60 million/mL | 84.8±18.1 | 70.8±28.2 | 80.3±22.2 | 0.31 | 83.5±22.1 | 68.8±34.7 | 60.1±32.9 | 0.02 |
| 100 million/mL | 86±9.6 | 77.3±25.4 | 79.4±20.8 | 0.52 | 93.1±4.1 | 76.7±17.8 | 66.9±29.9 | 0.007 |
| **Viability (%)** | **HA BG11**  **(n = 6)** | **HA Saline**  **(n = 6)** | **HA H_2_O**  **(n = 6)** | **P value** | **HA BG11**  **(n = 6)** | **HA Saline**  **(n = 6)** | **HA H_2_O**  **(n = 6)** | **P value** |
|  | **24 hours** | | | | **48 hours** | | | |
| Dark 30 million/mL | 72.3±24.5 | 38.4±33.5 | 76.7±15.7 | 0.05 | 52.5±32.3 | 26.5±31.7 | 75.4±28.7 | 0.004 |
| 60 million/mL | 58.5±37.4 | 59.9±37.1 | 61.2±26.2 | 1.00 | 49±37.5 | 40.4±22.1 | 51.8±31.7 | 0.37 |
| 100 million/mL | 66.3±40.7 | 55.9±40.6 | 61.8±31.0 | 0.79 | 52±25.4 | 64.1±32 | 52.1±27.4 | 0.3 |

HA, hyaluronic acid.

Data presented as mean ± standard deviation. P value compared three HA gel composition at 24 and 48 hours with and without light exposure. Statistical analyses performed using the analysis of variance.

**Table S4: Effect of *S. elongatus* concentration on oxygen production.**

| **O_2_ level (mmHg)** | **HA BG11**  **(n = 10)** | **HA Saline**  **(n = 10)** | **HA H_2_O**  **(n = 10)** | **P value** | **HA BG11**  **(n = 10)** | **HA Saline**  **(n = 10)** | **HA H_2_O**  **(n = 10)** | **P value** |
| --- | --- | --- | --- | --- | --- | --- | --- | --- |
|  | **6 hours** | | | | **18 hours** | | | |
| Light 30 million/mL | 156.4±9.8 | 146.5±3.1 | 144.6±3.7 | 0.003 | 173.5±13.6 | 143.2±3.5 | 138.3±5.1 | <0.0001 |
| 60 million/mL | 173.5±11.0 | 150.6±3.2 | 149.5±3.6 | <0.0001 | 198.7±4.1 | 145.3±3.4 | 145.1±6.9 | <0.0001 |
| 100 million/mL | 194.2±7.1 | 157.8±2.8 | 157.9±7.0 | <0.0001 | 200.0±0.0 | 161.4±14.8 | 160.3±9.1 | <0.0001 |
|  | **24 hours** | | | | **48 hours** | | | |
| Light 30 million/mL | 183.7±16.5 | 137.0±4.0 | 135.1±6.3 | <0.0001 | 187.2±13.6 | 138.7±4.0 | 137.6±4.5 | <0.0001 |
| 60 million/mL | 195.2±15.2 | 140.6±4.1 | 140.1±6.2 | <0.0001 | 188.3±17.4 | 141.9±3.7 | 139.7±3.5 | <0.0001 |
| 100 million/mL | 200.0±0.0 | 160.2±12.1 | 160.2±18.7 | <0.0001 | 200.0±0.0 | 145.0±6.1 | 145.6±8.2 | <0.0001 |
| **O_2_ level (mmHg)** | **HA BG11**  **(n = 6)** | **HA Saline**  **(n = 6)** | **HA H_2_O**  **(n = 6)** | **P value** | **HA BG11**  **(n = 6)** | **HA Saline**  **(n = 6)** | **HA H_2_O**  **(n = 6)** | **P value** |
|  | **6 hours** | | | | **18 hours** | | | |
| Dark 30 million/mL | 143.8±0.4 | 145.3±1.0 | 142.2±2.3 | 0.02 | 140.7±2.3 | 138.7±9.2 | 140.0±1.9 | 0.83 |
| 60 million/mL | 146.0±2.4 | 148.7±1.6 | 141.8±1.8 | <0.0001 | 143.3±1.6 | 136.8±10.3 | 137.5±1.8 | 0.21 |
| 100 million/mL | 148.0±1.3 | 149.2±1.5 | 145.3±4.1 | 0.03 | 140.0±2.4 | 137.3±6.0 | 138.5±2.7 | 0.43 |
|  | **24 hours** | | | | **48 hours** | | | |
| Dark 30 million/mL | 136.0±4.2 | 130.0±10.3 | 133.8±6.1 | 0.24 | 137.2±3.7 | 134.2±1.9 | 133.8±3.3 | 0.007 |
| 60 million/mL | 136.8±2.9 | 129.0±8.0 | 134.7±2.3 | 0.01 | 137.3±3.0 | 134.0±3.0 | 136.0±3.2 | 0.14 |
| 100 million/mL | 136.0±4.3 | 135.7±5.9 | 132.8±3.3 | 0.12 | 135.2±4.5 | 134.2±3.9 | 137.7±2.5 | 0.2 |

HA, hyaluronic acid.

Data presented as mean ± standard deviation. P value compared three HA gel composition at 24 and 48 hours at different *S. elongatus* concentration with and without light exposure. Statistical analyses performed using the analysis of variance.

**Table S5: *S. elongatus* viability over 5 weeks in the novel biologic gel in light.**

| **Viability (%)** | **24 Hours**  **(n = 5)** | **48 Hours**  **(n = 5)** | **1 Week**  **(n = 5)** | **2 Weeks**  **(n = 5)** | **3 Weeks**  **(n = 5)** | **4 Weeks**  **(n = 5)** | **5 Weeks**  **(n = 5)** |
| --- | --- | --- | --- | --- | --- | --- | --- |
|  | 90.7 ± 7.3 | 81.3 ± 16.9 | 89.4 ± 4.4 | 93.2 ± 3.6 | 76.2 ± 16.4 | 65.2 ± 32.4 | 39.1 ± 16 |

Data presented as mean ± standard deviation.

**Table S6: Human dermal fibroblast viability after treatment in the serum-starved, hypoxia condition for 24 hours with light exposure.**

| **Viability (%)** | **Before hypoxia**  **(n = 10)** | **Control**  **(n = 10)** | **HA Gel in BG11**  **(n = 10)** | **Biologic Gel**  **(n = 10)** | ***S. elongatus* in PBS**  **(n = 10)** |
| --- | --- | --- | --- | --- | --- |
|  | 94.9 ± 7.7 | 90.7 ± 11.6 | 78.4 ± 13.3 | 99.8 ± 0.3 | 86.8 ± 5.3 |
| P value compared to before hypoxia | - | 0.34 | 0.003 | 0.06 | <0.01 |
| P value compared to control | 0.34 | - | 0.04 | 0.02 | 0.34 |
| P value compared to biologic gel | 0.06 | 0.02 | <0.0001 | - | <0.0001 |

HA, hyaluronic acid.

Data presented as mean ± standard deviation. Biologic gel consisted of HA gel in BG11 with *S. elongatus* at 100 million cells/mL concentration. Statistical analyses were performed using the student t test.

**Table S7: Wound histology mean scoring from animals sacrificed 7 days after treatment using the rodent peripheral arterial disease burn wound model.**

| **Histology Features** | **Control**  **(n = 4)** | **Gel**  **(n = 4)** | **Biologic Gel in Light**  **(n = 6)** | **Biologic Gel in Dark**  **(n = 4)** |
| --- | --- | --- | --- | --- |
| Neutrophilic inflammation | 2.0 ± 1.8 | 3 ± 1.2 | 1.5 ± 1.0 | 1.0 ± 1.0 |
| Granulation tissue | 2.5 ± 1.3 | 4.0 ± 0.0 | 2.0 ± 0.9 | 1.3 ± 0.6 |
| Mononuclear inflammation | 2.5 ± 1.3 | 4.0 ± 0.0 | 2.0 ± 0.9 | 1.3 ± 0.6 |
| Tissue necrosis | 2.5 ± 2.1 | 2.5 ± 2.1 | 1.5 ± 1.0 | 1.7 ± 2.1 |
| Acanthosis and hyperkeratosis | 2.0 ± 1.2 | 2.3 ± 1.3 | 2.8 ± 1.2 | 4.0 ± 0.0 |

Data presented as median ± standard deviation. Tissue samples were scored from 0-4 based neutrophilic inflammation, granulation tissue, mononuclear inflammation, tissue necrosis, and acanthosis/hyperkeratosis with 0 being normal, 1 being ≤ 25% tissue affected, 2 being 25-50% tissue affected, 3 being 50-75% tissue affected, and 4 being ≥ 75% tissue affected.

**Table S8: Peripheral blood laboratory results of animals sacrificed after 7 and 14 days of treatment for both peripheral arterial disease wound models.**

| **Laboratory Test** | **Control** | **Gel** | **Biologic Gel in Light** | **Biologic Gel in Dark** |
| --- | --- | --- | --- | --- |

| Day 7 Peripheral Arterial Disease Burn Wound Model | | | | |
| --- | --- | --- | --- | --- |
|  | (n = 4) | (n = 4) | (n = 6) | (n = 3) |
| White blood cell (k/µL) | 11.0 ± 1.6 | 15.5 ± 2.6 | 13.3 ± 1.8 | 12.0 ± 2.5 |
| Platelet count (k/µL) | 1055.0 ± 69.7 | 862.5 ± 103.7 | 897.5 ± 107.3 | 913.0 ± 212.0 |
| Hematocrit (%) | 47.6 ± 2.2 | 46.7 ± 1.71 | 46.5 ± 0.8 | 46.7 ± 1.9 |
| Reticulocyte (%) | 3.0 ± 0.2 | 2.9 ± 0.9 | 2.6 ± 0.1 | 2.5 ± 0.1 |
| Neutrophil (%) | 29.8 ± 3.5 | 30.0 ± 4.0 | 23.0 ± 3.3 | 27.7 ± 2.0 |
| Lymphocyte (%) | 56.8 ± 6.1 | 61.0 ± 5.1 | 66.3 ± 4.5 | 62.7 ± 3.4 |
| Monocyte (%) | 10.8 ± 1.4 | 7.5 ± 1.5 | 8.2 ± 2.2 | 9 ± 2.1 |
| Eosinophil (%) | 0.5 ± 0.3 | 1.5 ± 0.7 | 2.3 ± 0.4 | 0.7 ± 0.3 |
| Basophile (%) | 0 | 0 | 0 | 0 |

| Day 14 Peripheral Arterial Disease Burn Wound Model | | | | |
| --- | --- | --- | --- | --- |
|  | (n = 3) | (n = 3) | (n = 3) | (n = 3) |
| White blood cell (k/µL) | 10.0 ± 2.3 | 9.9 ± 1.3 | 9.8 ± 0.0 | 11.1 ± 0.3 |
| Platelet count (k/µL) | 1269.3 ± 129.7 | 948.3 ± 88.7 | 1175.3 ± 181.4 | 1084.3 ± 59.8 |
| Hematocrit (%) | 47.1 ± 0.5 | 47.4 ± 1.7 | 47.7 ± 1.1 | 47.2 ± 0.4 |
| Reticulocyte (%) | 3.5 ± 0.4 | 4.0 ± 0.8 | 3.6 ± 0.3 | 4.1 ± 1.2 |
| Neutrophil (%) | 21.7 ± 4.7 | 29.7 ± 7.9 | 24.0 ± 5.6 | 20.0 ± 3.5 |
| Lymphocyte (%) | 69.7 ± 4.5 | 63.3 ± 7.8 | 68.3 ± 4.2 | 71.0 ± 4.6 |
| Monocyte (%) | 7.7 ± 1.7 | 6.7 ± 0.3 | 6.7 ± 1.2 | 8.3 ± 1.8 |
| Eosinophil (%) | 1.0 ± 0.6 | 0.3 ± 0.3 | 1.0 ± 0.6 | 0.7 ± 0.3 |
| Basophile (%) | 0.3 ± 0.3 | 0 | 0 | 0 |

| Day 7 Peripheral Arterial Disease Ischemic Wound Model |
| --- |

|  | (n = 5) | (n = 4) | (n = 5) | (n = 5) |
| --- | --- | --- | --- | --- |
| White blood cell (k/µL) | 10.8 ± 0.5 | 12.3 ± 0.6 | 9.3 ± 12.5 | 10.9 ± 0.6 |
| Platelet count (k/µL) | 1251.8 ± 85.8 | 1094.8 ± 169.7 | 1020.6 ± 252.4 | 1395.0 ± 80.5 |
| Hematocrit (%) | 45.9 ± 0.6 | 48.0 ± 0.6 | 45.7 ± 1.0 | 44.8 ± 0.7 |
| Reticulocyte (%) | 3.8 ± 0.3 | 4.1 ± 0.4 | 3.5 ± 0.2 | 4.9 ± 0.6 |
| Neutrophil (%) | 18.0 ± 2.1 | 19.2 ± 3.4 | 22.4 ± 2.9 | 16.0 ± 1.9 |
| Lymphocyte (%) | 72.2 ± 2.2 | 70.6 ± 3.8 | 67.4 ± 2.6 | 71.6 ± 2.1 |
| Monocyte (%) | 9.0 ± 1.5 | 9.4 ± 1.4 | 8.4 ± 2.0 | 10.8 ± 1.6 |
| Eosinophil (%) | 0.8 ± 0.8 | 0.8 ± 0.5 | 1.8 ± 0.5 | 1.6 ± 0.7 |
| Basophile (%) | 0 | 0 | 0 | 0 |

| Day 14 Peripheral Arterial Disease Ischemic Wound Model |
| --- |

|  | (n = 5) | (n = 4) | (n = 5) | (n = 4) |
| --- | --- | --- | --- | --- |
| White blood cell (k/µL) | 10.6 ± 1.7 | 11.3 ± 3.0 | 12.2 ± 1.3 | 9.9 ± 1.4 |
| Platelet count (k/µL) | 1235.0 ± 54.2 | 718.0 ± 233.2 | 919.4 ± 52.5 | 713.3 ± 225.0 |
| Hematocrit (%) | 45.0 ± 0.7 | 40.3 ± 6.4 | 49.0 ± 0.6 | 45.0 ± 1.1 |
| Reticulocyte (%) | 5.0 ± 0.7 | 4.6 ± 0.6 | 5.0 ± 0.4 | 4.0 ± 0.2 |
| Neutrophil (%) | 24.4 ± 4.1 | 25.8 ± 1.9 | 36.0 ± 5.6 | 22.3 ± 2.7 |
| Lymphocyte (%) | 64.4 ± 5.3 | 68.3 ± 2.8 | 58.0 ± 5.2 | 70.8 ± 2.3 |
| Monocyte (%) | 9.0 ± 1.9 | 4.5 ± 1.0 | 5.4 ± 0.4 | 6.5 ± 1.0 |
| Eosinophil (%) | 2.2 ± 0.5 | 1.5 ± 0.5 | 0.6 ± 0.4 | 0.5 ± 0.3 |
| Basophile (%) | 0 | 0 | 0 | 0 |

Data presented as mean ± standard error.

For the peripheral arterial disease burn wound model, 1 animal treated with biologic gel in dark sacrificed on day 7, 1 control animal sacrificed on day 14, and 1 animal treated with biologic gel in light sacrificed on day 14 were unable to have peripheral blood laboratory results analyzed due to clotted samples. For the peripheral arterial disease ischemic wound model, 1 animal treated with gel sacrificed on day 7, 1 animal treated with biologic gel in dark sacrificed on day 14, and another animal treated with gel sacrificed on day 14 were unable to have peripheral blood laboratory results analyzed due to clotted samples.
